# Drug-loaded microbubble delivery system to enhance PD-L1 blockade immunotherapy with remodeling immune microenvironment

**DOI:** 10.1186/s40824-023-00350-5

**Published:** 2023-02-09

**Authors:** Jun Zheng, Ju Huang, Liang Zhang, Mengna Wang, Lihong Xu, Xiaoyun Dou, Xiaojing Leng, Mingxiao Fang, Yang Sun, Zhigang Wang

**Affiliations:** 1 State Key Laboratory of Ultrasound in Medicine and Engineering, Institute of Ultrasound Imaging, The Second Affiliated Hospital, Chongqing Medical University, Chongqing, 400010 People’s Republic of China; 2 Ultrasound Department, The First Affiliated Hospital of Chongqing Medical University, Chongqing, 400042 People’s Republic of China; 3 Department of Pathology, College of Basic Medicine, Chongqing Medical University, Chongqing, 400016 People’s Republic of China; 4 Institute of Life Sciences, Chongqing Medical University, Chongqing, 400016 People’s Republic of China

**Keywords:** Ultrasound-targeted microbubble destruction (UTMD), Immune checkpoint blockade (ICB), Docetaxel (DTX), PD-L1, Tumor immunosuppressive microenvironment

## Abstract

**Background:**

Although programmed cell death protein 1 (PD-1)/ programmed cell death-ligand protein 1 (PD-L1) checkpoint blockade immunotherapy demonstrates great promise in cancer treatment, poor infiltration of T cells resulted from tumor immunosuppressive microenvironment (TIME) and insufficient accumulation of anti-PD-L1 (αPD-L1) in tumor sites diminish the immune response. Herein, we reported a drug-loaded microbubble delivery system to overcome these obstacles and enhance PD-L1 blockade immunotherapy.

**Methods:**

Docetaxel (DTX) and imiquimod (R837)-loaded microbubbles (RD@MBs) were synthesized via a typical rotary evaporation method combined with mechanical oscillation. The targeted release of drugs was achieved by using the directional "bursting" capability of ultrasound-targeted microbubble destruction (UTMD) technology. The antitumor immune response by RD@MBs combining αPD-L1 were evaluated on 4T1 and CT26 tumor models.

**Results:**

The dying tumor cells induced by DTX release tumor-associated antigens (TAAs), together with R837, promoted the activation, proliferation and recruitment of T cells. Besides, UTMD technology and DTX enhanced the accumulation of αPD-L1 in tumor sites. Moreover, RD@MBs remolded TIME, including the polarization of M2-phenotype tumor-associated macrophages (TAMs) to M1-phenotype, and reduction of myeloid-derived suppressor cells (MDSCs). The RD@MBs + αPD-L1 synergistic therapy not only effectively inhibited the growth of primary tumors, but also significantly inhibited the mimic distant tumors as well as lung metastases.

**Conclusion:**

PD-L1 blockade immunotherapy was enhanced by RD@MBs delivery system.

**Supplementary Information:**

The online version contains supplementary material available at 10.1186/s40824-023-00350-5.

## Introduction

In recent years, immune checkpoint (*e.g.*, programmed cell death protein 1 (PD-1)/ programmed cell death-ligand protein 1 (PD-L1)) blockade (ICB) therapy has emerged as a promising therapeutic strategy against cancer [1–3]. However, only less than 30% of the patients showed antitumor immune response to PD-1/PD-L1 ICB [4]. Accumulating evidence has demonstrated that PD-1/PD-L1 blockade therapy relies on infiltration of tumor-specific cytotoxic T lymphocytes (CTLs) and accumulation of the therapeutic antibodies at tumor sites [5, 6]. However, immune suppression caused by immunosuppressive cells (*e.g.*, tumor-associated macrophages (TAMs), regulatory T cells (Treg) and myeloid-derived suppressor cells (MDSCs)) in tumor microenvironment (TME) limit the activation and proliferation of CTLs [7].

TAMs, which frequently make up a substantial proportion of all tumor-infiltrating immune cells, can typically polarize into M1 or M2-phenotype in response to different microenvironmental stimulations [7, 8]. M1-phenotype TAMs are known to kill tumor cells by producing various pro-inflammatory cytokines (*e.g.*, IL-12 and TNF-α) and inducible nitric oxide synthase (iNOS). On the other hand, M2-phenotype TAMs facilitate abnormal angiogenesis by producing high levels of vascular endothelial growth factor (VEGF) and matrix metalloproteinase-9 (MMP9), which further limits recruitment of CTLs [4, 9, 10]. As another immunosuppressive cell, Tregs promote tumor cell proliferation by inhibiting antigen-presenting cells (APCs), depleting key cytokines that activate effector T cells, and produce immunosuppressive humoral factors, leading to the tumor immunosuppressive microenvironment (TIME) and inhibition of CTLs activation [11]. As a phenotypically heterogeneous cell population, MDSCs induce T cell dysfunction and reduce its proliferation, thereby promoting CTLs apoptosis [12]. Therefore, regulating the expression of PD-L1 on the tumor cells and alleviating the TIME to promote the infiltration of CTLs are critical to improve the response rate of PD-1/PD-L1 ICB therapy.

To strengthen the therapeutic efficiency, many immune adjuvants, including Toll-like receptor (TLR) agonists, have been used in tumor immunotherapy [7, 13]. Such immune adjuvants promote the recruitment of antigen-specific CD8^+^ T cells to tumor areas by stimulating dendritic cells (DCs) maturation and cytokine secretion [14]. Studies have reported that agonists of TLR7/TLR8/TLR9 can induce the polarization of M2-phenotype TAMs to M1-phenotype through the nuclear factor-κB (NF-κB) signaling pathway [15–17]. Despite desirable performance of TLR agonists, systemic exposure to TLR agonists, will activate immune cells in a non-discriminative manner, which will inevitably lead to an excessive immune response and even damage to normal tissues [18]. Therefore, unregulated administration of immune adjuvants is highly risky, and an on-demand release of the drug in the tumor area is extremely important.

Many efficient drug delivery systems have been developed to enhance drug penetration and delivery precision [19]. Among them, ultrasound-targeted microbubble destruction (UTMD) technology was reported to achieve safe and efficient drug delivery [20]. After intravenous injection of ultrasonic microbubbles, these microbubbles will oscillate and collapse where an ultrasound irradiation is applied, thereby enabling a localized acoustic cavitation effect [21]. In the meantime, the sonoporation produced by mechanical microfluidics and shear stress during microbubble destruction can enhance local vascular permeability and increase drug penetration [22], which would aslo promote the accumulaiton of therapeutic antibodies.

Chemotherapy is one of the most common interventions against cancer. A number of clinical studies have shown that chemotherapy combined with ICB therapy can significantly reduce tumor progression and prolong survival in cancer patients by stimulate specific immune responses. As a first-line chemotherapeutic drug, docetaxel (DTX) can effectively remodel the TIME and enhance the antitumor immune response [23]. In addition, studies have shown that chemotherapy can further upregulate the expression of PD-L1 in tumor cells, which can enhance the accumulation of αPD-L1 in tumor to improve the response rate of ICB therapy [24, 25]. Therefore, DTX-based chemotherapy combined with ICB therapy is expected to become a novel and more effective way for tumor combinational therapy.

**Scheme 1:**
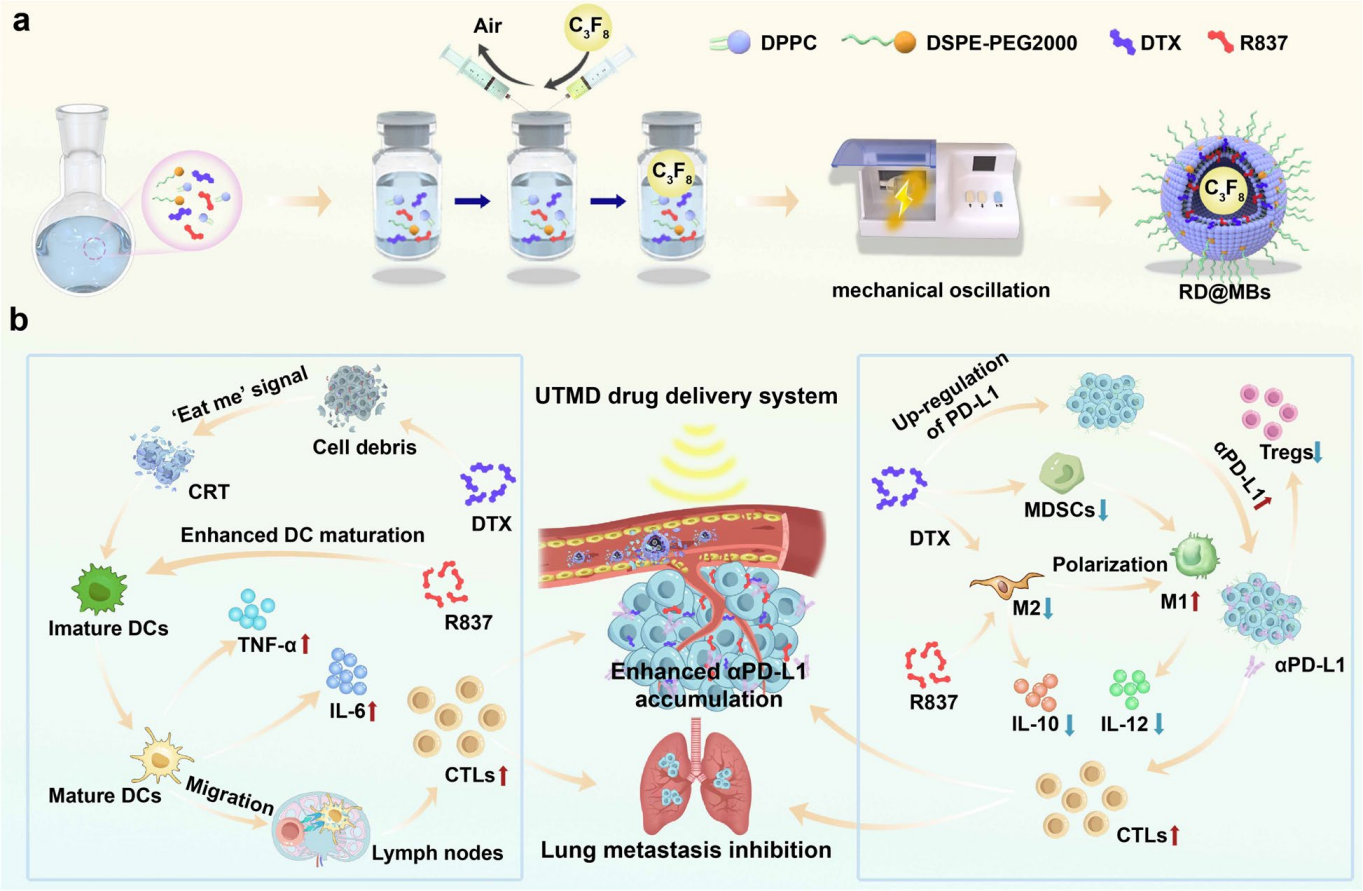
Schematic illustration of the preparation of RD@MBs and antitumor immune responses. **a** Schematic diagram of the preparation process for RD@MBs. **b** Schematic diagram of the mechanism of in vivo antitumor immune response induced by RD@MB combined with immune checkpoint blockade therapy

In this work, we constructed a UTMD drug delivery system for enhanced anti-PD-L1 (αPD-L1) blockade immunotherapy (Scheme 1a). As showed in Scheme 1b, in this system, chemotherapeutic drug DTX and immune adjuvant R837 are loaded into MBs, and the targeted release of drugs is achieved by using the directional “bursting” capability of UTMD technology. The released DTX would attack cancer cells and cause cell death, the dying cells could release tumor-associated antigens (TAAs). These antigens, along with R837, would effectively promote the maturation of DCs, accomplished effectively with pro-inflammatory cytokines secretion including interleukin-6 (IL-6) and tumor necrosis factor-α (TNF-α). Mature DCs present antigens to naïve T cells, and induce them to be CTLs. DTX and R837 will alleviate the TIME by reducing MDSCs and inducing polarization of M2-phenotype TAMs towards M1-phenotype, which will decrease the secretion of anti-inflammatory cytokine interleukin-10 (IL-10) and increases pro-inflammatory cytokine interleukin-12 (IL-12). The combination of αPD-L1 reduced Treg cells in the TIME and inhibited the tumor growth and metastasis. Moreover, UTMD technology as well as DTX, which would upregulate PD-L1 expression on tumor cells, can promote the intertumoral accumulation of αPD-L1. Therefore, this UTMD drug delivery system greatly improves tumor response to αPD-L1 blockade immunotherapy by remodeling the TIME and promoting intertumoral accumulation of αPD-L1.

## Materials and methods

### Materials and regents

Lipids including 1,2-Dipalmitoyl-sn-glycero-3-phosphocholine (DPPC) and 1,2-distearoyl-sn-glycero-3-phosphoethanolamine-N-[methoxy(polyethylene glycol)-2000] (DSPE-mPEG2000) are analytically pure and were purchased from Xi’an Ruixi Biological Technology Co., Ltd. (Xi’an, China); Imiquimod (R837) and docetaxel (DTX) are analytically pure and were purchased from Macklin Biochemical Co., Ltd. (Shanghai, China). Glycerol was purchased from Sigma-Aldrich (St. Louis, USA). Anti-mouse PD-L1 antibody (αPD-L1, Clone: 10 F.9G2, Catalog No. BE0101) was purchased from BioXcell (West Lebanon, New Hampshire). High glucose Dulbecco’s modified Eagle’s medium (DMEM) and Fetal bovine serum (FBS) were purchased from Gibco Invitrogen. Penicillin–streptomycin (1%, PS) and 0.25% trypsin–EDTA were purchased from Beyotime (Shanghai, China). FIX & PERM Kit was purchased from MultiSciences Biotech Co., Ltd, Hangzhou, China. The following antibodies were used: PE-conjugated anti-mouse CD86 (clone GL1, REF. NO. 12–0862-82, eBioscience), APC-conjugated anti-mouse CD80 (clone 16-10A1, REF. NO. 17–0801-82, eBioscience), FITC-conjugated anti-mouse CD11c (clone N418, REF. NO. 11–0114-82, eBioscience), FITC-conjugated anti-mouse Gr-1 (clone RB6-8C5, REF. NO. 11–5931-82, eBioscience), APC-conjugated anti-mouse CD206 (clone MR6F3, REF. NO. 17–2061-82, eBioscience), PerCP-CY5.5-conjugated anti-mouse CD11b (clone M1/70, REF. NO. 45–0112-82, eBioscience), FITC-conjugated anti-mouse CD4 (clone GK1.5, REF. NO. 11–0041-82, eBioscience), APC-conjugated anti-mouse Foxp3 (clone FJK-16 s, REF. NO. 17–5773-82, eBioscience), APC-conjugated anti-mouse CD8a (clone 53–6.7, REF. NO. 100712,biolegend), PE-conjugated anti-mouse CD3 (clone 17A2, REF. NO. 12–0032-82, eBioscience), FITC-conjugated anti-mouse F4/80 (clone BM8, REF. NO. 11–4801-82, eBioscience), Anti-Calreticulin (ab92516, abcam), Goat Anti-Rabbit IgG H&L(FITC) (ab7086,abcam). ELISA kits for Mouse IL-6, IL-10, IL-12, TNF-α and IFN-γ were purchased from Meimian (Jiangsu, China).

### Synthesis of RD@MBs

RD@MBs were synthesized via a typical rotary evaporation method combined with mechanical oscillation. Briefly, R837 (6 mg), DTX (8 mg), DPPC (20 mg) and DSPE-PEG2000 (8 mg) were firstly dissolved in trichloromethane (10 mL). The following evaporations (-0.1 Mpa, 100 rpm, 55 °C, 1 h) were performed on a rotary evaporator (RE-52, Shanghai, China). Next, 1.8 mL phosphate-buffered saline (PBS) and 0.2 mL glycerol were added to the obtained lipid film, which were then peeled assisted by bath sonication. Add the resulting emulsion (0.5 mL) to a vial and replace the air in the vial with C_3_F_8_ gas using a gas exchange system. Finally, the vial was mechanical shaken using a dental amalgamator (YJT-2, Shanghai, China). The mixture was centrifugated (300 × g, 3 min) and the liquid was discarded to obtain purified MBs.

### Characterizations of RD@MBs

The morphology of RD@MBs was observed using an optical microscope (Leica, Germany). The loading efficiency of R837 and DTX in the MBs were evaluated by Liquid chromatography–mass spectrometry (LC–MS, TSQ Quantum and UltiMate 3000 RS, Thermo Scientific, USA).

### Cell culture and animals

The 4T1 murine breast cancer cell line and DC2.4 murine dendritic cell line (immature dendritic cells) were originally obtained from Zhongqiaoxinzhou Biotechnology Co., Ltd. (Shanghai, China) and RAW264.7 murine monocyte macrophage leukemia cell line (M0-phenotype TAMs) and CT26.WT murine colorectal cancer cell line was purchased from Procell Life Science&Technology Co., Ltd. (Wuhan, China). The cells cultured with the recommended medium at 37 °C in a 5% CO_2_ incubator.

Female BALB/c mice (6–8 weeks) were purchased from Chongqing Medical University and all in vivo experiments were approved by the Animal Experimentation Ethics Committee of The First Affiliated Hospital of Chongqing Medical University. Mice were housed in the Laboratory Animal Center of Chongqing Medical University under specific conditions with unlimited access to food and water. To establish a 4T1 orthotopic murine breast-cancer model, 4T1 cells (1 × 10^6^) were injected into the mammary fat pads of mice on the right side. Four days later, the same number of cells were injected into the left mammary fat pads of the mice to establish a bilateral tumor model.

### Biosafety assay

To investigate the biosafety of RD@MBs in vitro, cells were seeded into 96-well plates (5 × 10^3^ per well) and cultured overnight, and RD@MBs were added at different concentrations (phospholipid concentrations ranged from 50 to 1000 μg/mL), followed by incubation for another 24 h. Cell viabilities were evaluated by CCK-8 assay based on the absorbance at the wavelength of 450 nm by using an SpectraMax Paradigm Multi-Mode Microplate Reader (Molecular Devices, Shanghai, China). Subsequently, different concentrations (0 mg/mL, 3.5 mg/mL and 7 mg/mL at 200 μL) of RD@MBs were intravenously injected into healthy female BALB/c mice. On the 30^th^ day, the blood of mice was collected for blood biochemical and blood routine analyses. Untreated healthy mice were used as controls. Meanwhile, the major organs (heart, liver, spleen, lung, and kidney) of mice were collected and then subjected to H&E staining for histological analysis.

### UTMD Drug delivery system

RD@MBs at different concentrations (20, 40, 60, 80 and 100 μg/mL) were placed in agarose gel holes and US images in B-mode and Contrast mode were acquired with Vevo LAZR System (21 MHz, VisualSonics, Inc., Canada). For in vivo US imaging, tumor-bearing mice were intravenously injected with RD@MBs (3.5 mg/mL, 200 μL), and the US images of the tumor regions were acquired. The corresponding echo intensity was analyzed using an US imaging analysis software.

To invest drug delivery efficiency of UTMD technology**,** tumor-bearing mice were randomly divided into three groups including (1) free DiI, (2) DiI@MBs and DiI@MBs + US. The corresponding MBs (3.5 mg/mL, 200 μL) were intravenously administrated and the 3^rd^ group tumors were exposed to US irradiation immediately (1.0 MHz, 1.5 W/cm^2^, 50% duty cycle, 3 min). Tumors were dissected and analyzed using FCM. A total of 10,000 events were collected for each sample.

### Cellular PD-L1 expression and uptake of Cy5.5-αPD-L1

For detecting PD-L1 expression in vitro, 4T1 cells were seeded in confocal dishes to grow overnight. Subsequently, the cells were treated with different microbubbles (R@MBs, D@MBs, RD@MBs, 250 μg/mL, MBs were destroyed by US irradiation in advance, and the in vitro experiments involved in this study were treated with the same method) and cocultured for 24 h. The cells were fixed with 100 μL of 4% paraformaldehyde for 10 min and stained with the first antibody: anti–PD-L1 antibody (EPR20529, Abcam) (1 μg/mL, 100 μL) overnight at 4 ℃. The cells were stained with secondary antibody FITC–conjugated IgG for another 1 h. Cell nucleus were stained blue with DAPI and observed by CLSM. The same protocol was used for flow cytometry (FCM) analysis. Western blot was performed to further study PD-L1 expression. In detail, 4T1 cells were treated as mentioned above and the lysates of 4T1 cells were collected by cytoplasmic protein extraction kit (Servicebio, Wuhan, China). The lysates of 4T1 cells for western blotting analysis were incubated with different primary antibodies (anti-PD-L1 antibody and anti-Vinculin antibody (EPR20407)).

To evaluate the uptake of Cy5.5-αPD-L1, 4T1 cells were seeded in confocal dishes and treated with RD@MBs (250 μg/mL). Untreated cells were used as control. Then replaced with containing Cy5.5-αPD-L1 medium. After incubation for different times (2, 4, 6, 8 h) at 37℃, the cells were observed by CLSM. For FCM experiments, the 4T1 cells were seeded in 12-well plates and treated with RD@MBs. After incubated with Cy5.5-αPD-L1 for different amounts of time, the cells were harvested and analyzed using FCM.

### In vitro immune-system activation

To assess antitumor activity in vitro, cells were seeded into 96-well plates (5 × 10^3^ per well), cultured overnight, and then treated with different concentrations of R@MBs, D@MBs, RD@MBs (ranging from 50 to 1000 μg/mL) and cocultured for 24 h. Cell viabilities were evaluated by a CCK-8 assay. Furthermore, the dead and live cells were distinguished by confocal laser scanning microscopy (CLSM) observation after Calcein-AM/PI staining.

To study the release of CRT induced by DTX, the 4T1 cells were seeded in confocal dishes cultured overnight. Each sample treated with different MBs (PBS, R@MBs, D@MBs, RD@MBs, 250 μg/mL) and cocultured for 24 h. The cells were fixed and stained with anti–CRT antibody (EPR3924, Abcam) (1 μg/mL, 100 μL) for 1 h. After that, the cells were stained with secondary antibody FITC–conjugated IgG for another 1 h. Cell nucleus were stained by DAPI and observed by CLSM.

For in vitro DC stimulation experiments, transwell plates (Corning3422, USA) were used to co-incubate DC2.4 cells and treated-4T1 cells. 4T1 cells were seeded into another 12-well plates and treated as mentioned above (PBS, R@MBs, D@MBs, RD@MBs, 250 μg/mL). These treated-4T1 cells were harvested via centrifugation and seeded into the upper chamber while DC2.4 cells were seeded into the lower chamber of transwell system. After 24 h of co-incubation, DC2.4 cells were collected and stained with anti-CD11c FITC, anti-CD86 PE, anti-CD80 APC. FCM was used to analyze the ratio of CD80^+^ CD86^+^ CD11C^+^ DCs. The proinflammatory cytokines (*i.e.*, IL-6 and TNF-α) from DC suspension were tested by using enzyme-linked immunosorbent assay (ELISA) kits following a standard protocol.

### In vitro macrophages polarization

To verify the polarization of M0-TAMs to M1-type, RAW264.7 cells were seeded in 12-well plates. After adherence, they were divided into four groups: PBS, R@MBs, D@MBs, and RD@MBs (the concentration of microbubbles was 250 μg/mL). Next, in order to verify the polarization of M2-TAMs to M1-type, RAW264.7 cells were first induced to M2-type macrophages. RAW264.7 macrophages were inoculated in 12-well plates 5× 10^5^ cells until adherent, the medium was then removed, replaced with IL-4 (20 ng/mL) and IL-13 (20 ng/mL). After culturing for 24 h, different microbubbles (PBS, R@MBs, D@MBs, RD@MBs, 500 μg/mL) were added. After 24 h of incubation, the cells were collected by centrifugation, stained with anti-CD86 PE and anti-CD206 APC, and the phenotypic changes of M1 and M2 macrophages were observed by FCM, and the cytokines IL-10 and IL-12 in the cell supernatant were determined by ELISA.

### In vivo experiment

Bilateral 4T1-tumor models were established to investigate therapeutic efficacy of chemoimmunotherapy. The day of right mammary fat pad injection was defined as day -7, and the day of injection in the left was regarded as day -4. On day 0, mice were randomly divided into 7 treatment groups including (1) PBS, (2) D@MBs, (3) αPD-L1, (4) R@MBs + αPD-L1, (5) D@MBs + αPD-L1, (6) RD@MBs and (7) RD@MBs + αPD-L1. The corresponding MBs (3.5 mg/mL, 200 μL) were intravenously administrated and tumors were exposed to US irradiation immediately (1.0 MHz, 1.5 W/cm^2^, 50% duty cycle, 3 min). αPD-L1 (30 μg/ per mouse) was injected intravenously in mice on the 1^st^, 4^th^ and 7^th^ days. Tumor volumes and body weights were monitored every 2 days for 18 days. Tumor volume was calculated by following the formula: volume = width^2^ × length/2. Mice were euthanized when tumor volume reached 1000 mm^3^. Mice were subjected to survival analysis and observed until day 40. The lung tissue was removed after the euthanasia, then fixed and stained with Bouin's fluid to observe the lung metastasis of the mice in each group.

To study immune activation in vivo, tumor-bearing mice were randomly divided into 4 groups including (1) PBS, (2) R@MBs, (3) D@MBs and (4) RD@MBs. The corresponding MBs (3.5 mg/mL, 200 μL) were intravenously administrated and tumors were exposed to US irradiation. On the 3^rd^ day, the spleens, primary tumors and draining lymph nodes were dissected and single cell suspensions were prepared to study the DC maturation rate. The single cell suspensions were stained with anti-CD11c FITC, anti-CD86 PE and anti-CD80 APC for FCM analysis. In the meantime, the serum concentration of IL-6 and TNF-α were measured using ELISA. To monitor the macrophage polarization, the spleens and tumors of mice were collected to prepare single cell suspensions on the 8^th^ day. Surface expression of macrophage lineage markers was quantified by FCM after staining with anti-CD11b PerCP-CY5.5, anti-F4/80 FITC, anti-CD86 PE and anti-CD206 APC. The single cell suspensions were also stained with anti-Gr-1 FITC and anti-CD11b PerCP-CY5.5 to study the papulation of MDSCs. The levels of IL-10 and IL-12 in serum were measured by ELISA. In addition, to demonstrate the expression of CTLs and Treg, the mice were randomly divided into 7 groups: (1) PBS, (2) D@MBs, (3) αPD-L1, (4) R@MBs + αPD-L1, (5) D@MBs + αPD-L1, (6) RD@MBs and (7) RD@MBs + αPD-L1. On the 8^th^ day, the spleen and metastases were collected. Single cell suspension was prepared, and stained with anti-CD3 PE, anti-CD4 FITC, anti-CD8 APC and anti-CD3 PE, anti-CD4 FITC, and anti-FOX P3 APC to study the infiltration of CTLs (CD3^+^CD4^−^CD8^+^ as the marker) and Treg (CD3^+^CD4^+^FOXP3^+^ as the marker) in systemic and metastatic tumors.

### Statistical analysis

The data measured in this experiment were all expressed as mean ± standard deviation (SD). The data was analyzed by Student’s *t*-test to determine the significant differences between two groups. *p < 0.05, **p < 0.01, ***p < 0.001.

## Results

### Synthesis and characterization of RD@MBs

As US imaging contrast agents, MBs (SonoVue, Definity, Optison, etc*.*) have been widely used clinically. Lipid RD@MBs were prepared by the process shown in Scheme 1a. The hydrophobic immune adjuvant R837 and chemotherapeutic drug DTX were loaded into the lipid shell of the MBs, and the core was filled with perfluoropropane (C_3_F_8_), which prolongs the lifetime of MBs during blood circulation due to its low solubility and low diffusion coefficient [26]. The prepared RD@MBs dispersed in PBS were milky white in appearance (Fig. S1a, inset) with good dispersion, and the RD@MBs showed a uniform spherical shape under the optical microscope (Fig. S1a). The standard curves of DTX (Fig. S1b) and R837 (Fig. S1c) were plotted by LC–MS assay, and the encapsulation efficiencies were calculated to be 72.90% and 65.02%, respectively.

### Biosafety assay

Good biosafety is the primary prerequisite for advanced applications of MBs. The cytotoxic effect of different concentrations of RD@MBs (50 μg/mL, 100 μg/mL, 250 μg/mL, 500 μg/mL and 1000 μg/mL) on 4T1 cells without US irradiation was determined by a standard CCK-8 assay. After incubation with different concentrations of RD@MBs, the cell viabilities were all higher than 90%, and showed no significant toxicity to 4T1 cells, indicating the high biosafety of RD@MBs (Fig. S2a). It also indicated that in the absence of ultrasound irradiation, the MBs will not release the drug effectively, enabling tumor-site-specific treatment without affecting normal tissue. To further investigate the in vivo biosafety of RD@MBs, healthy mice were administrated with RD@MBs at the dosage we would use in the following experiment and at the double dosage. After 30 days, compared with the control group, the renal function indexes, cardiac function indexes, liver function indexes and blood routine indexes of the treated mice showed neglectable fluctuation even after doubling the dose (Fig. S2b). In addition, no overt pathologic changes were observed in the main organs (heart, liver, spleen, lung, kidney) (Fig. S2c). These results demonstrated the good biocompatibility of RD@MBs, laying the foundation for the following in vivo applications.

### UTMD Drug delivery system

In order to determine the time window of US irradiation after RD@MBs injection, it is important to investigate the perfusion of MBs in the tumor area. First, in vitro US imaging ability of RD@MBs was evaluated. As shown in Fig. S3a, the US intensity gradually increased with the elevation of the MBs concentration, indicating the superior US imaging enhancement capability. US intensity in the region of interest was measured and plotted against concentrations of RD@MBs (Fig. S3b). The US intensities in both B mode and CEUS mode correlate linearly with concentrations of RD@MBs. Subsequently, RD@MBs were intravenously injected into 4T1 tumor-bearing mice, and the perfusion of RD@MBs in the tumor tissues was observed by CEUS imaging. As shown in Fig. S3c and d, obvious perfusion could be observed in the tumor area 15 s after injection of RD@MBs. The perfusion gradually decreased with prolonged time, and almost completely faded at approximately 5 min. According to the results, US irradiation was performed immediately after intravenous injection of RD@MBs to achieve drug accumulation in the tumor areas.

To demonstrate the drug delivery efficiency of the UTMD technology, the mean fluorescence intensity (MFI) of the tumor was analyzed using the MBs labeled with DiI. As shown in Fig. S4a and b, the MFI in DiI@MBs + US group was significantly higher than that in free DiI and DiI@MBs groups, indicating that US irradiation enhances the release of drugs at the tumor site and greatly improves the concentration of drugs in the tumor.

### PD-L1 Expression and cellular uptake of αPD-L1

PD-1/PD-L1 ICB immunotherapy is based on PD-L1 expression in tumor tissue. Tumor PD-L1 expression is considered a predictive biomarker that can be used to pinpoint potential candidates who may benefit from PD-1/PD-L1 ICB immunotherapy [27]. Recent studies have documented that chemotherapy can upregulate PD-L1 expression in tumor cells [28], and that the increased PD-L1 expression could promote the uptake of αPD-L1, which further improves the response rate to ICB. Therefore, the effect of DTX on PD-L1 expression in tumor cells and the uptake of αPD-L1 were studied next.

**Fig. 1:**
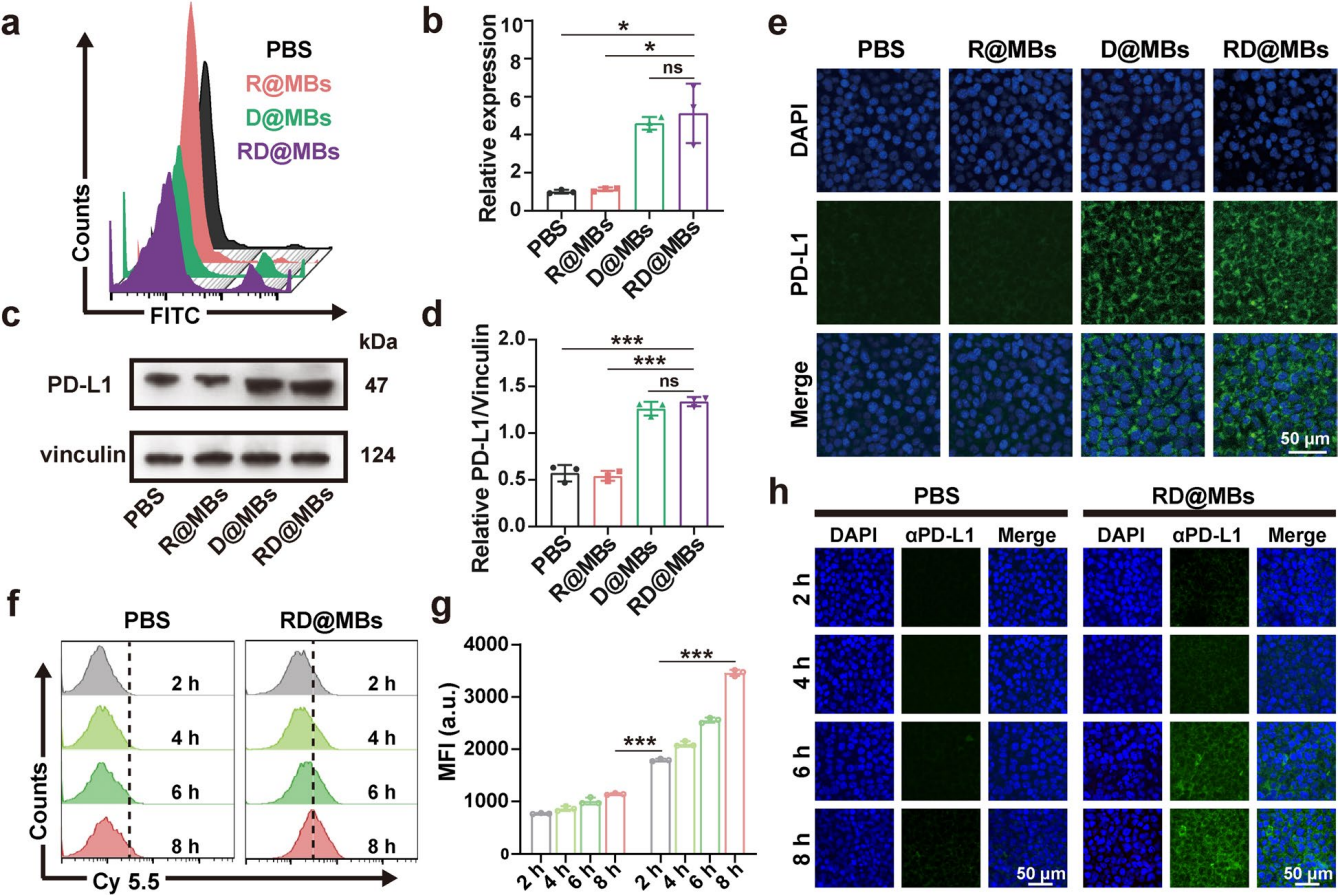
Increased PD-L1 expression and cellular uptake in tumor cells after DTX stimulation. **a**-**b** FCM results of the PD-L1 expression on 4T1 cells after various treatments (PBS, R@MBs, D@MBs, RD@MBs) and quantification of the relative expression. **c**-**d** Western blot analysis of the relative expression of PD-L1 on tumor cells. **e** Representative CLSM images of PD-L1 immunofluorescence staining of 4T1 cells. Blue, DAPI-labeled nucleus; green, FITC–conjugated anti-PD-L1 antibody-labeled PD-L1 (scale bar = 50 μm). **f**-**g** FCM results of cellular uptake of Cy5.5-labeled αPD-L1 after PBS and RD@MBs treatments and the corresponding quantitative analysis of MFI. **h** CLSM images of cellular uptake of Cy5.5-labeled αPD-L1. Blue, DAPI-labeled nucleus; green, Cy 5.5-labeled αPD-L1 (scale bar = 50 μm). Data are expressed as mean ± SD (*n* = 3). Statistical significances were calculated via Student’s t test, *p < 0.05, **p < 0.01 and ***p < 0.001

The expression of PD-L1 on tumor cells after DTX treatment in vitro was analyzed. FCM analysis showed that the expression of PD-L1 on cells treated with DTX-containing D@MBs and RD@MBs was significantly increased (Fig. 1a), with relative expression levels at 4.59 ± 0.33 and 5.13 ± 1.56, respectively, the relative expression in the R@MBs group was 1.12 ± 0.10, which was not significantly different from the control group (Fig. 1b). Western blot and immunofluorescence staining showed similar results. Western blot results showed that D@MBs and RD@MBs-treated cells expressed more PD-L1 than PBS and R@MBs-treated cells, and their relative protein expression was approximately twice that of control and R@MBs (Fig. 1c, d). Similarly, the most obvious green fluorescence, which represents PD-L1, was observed in the DTX-contained D@MBs and the RD@MBs groups when the cells were subjected to immunofluorescence staining (Fig. 1e). These results demonstrate that DTX can significantly upregulate the expression of PD-L1 on tumor cells.

Next, we investigated the effect of PD-L1 upregulation on αPD-L1 uptake using Cy5.5-labeled αPD-L1. FCM analysis showed that the uptake of αPD-L1 was higher than that in the RD@MBs group, and MFI gradually increased over time (Fig. 1f, g). In marked contrast, there was only a small amount of Cy5.5-αPD-L1 uptake in the control group, and the uptake of Cy5.5-αPD-L1 was not significantly increased with time. Similar results were also observed in CLSM, where the fluorescence of the RD@MBs group was significantly stronger than that of the control group and its fluorescence intensity gradually increased (Fig. 1h). Overall, in addition to acting as a conventional chemotherapeutic drug, DTX can upregulate the expression of PD-L1 in tumor cells, providing a more effective target for ICB therapy, and promote the uptake of αPD-L1. Therefore, it can be expected that the combined use of DTX and αPD-1 will greatly improve the response rate of ICB.

### RD@MBs mediated immune-system activation in vitro

**Fig. 2:**
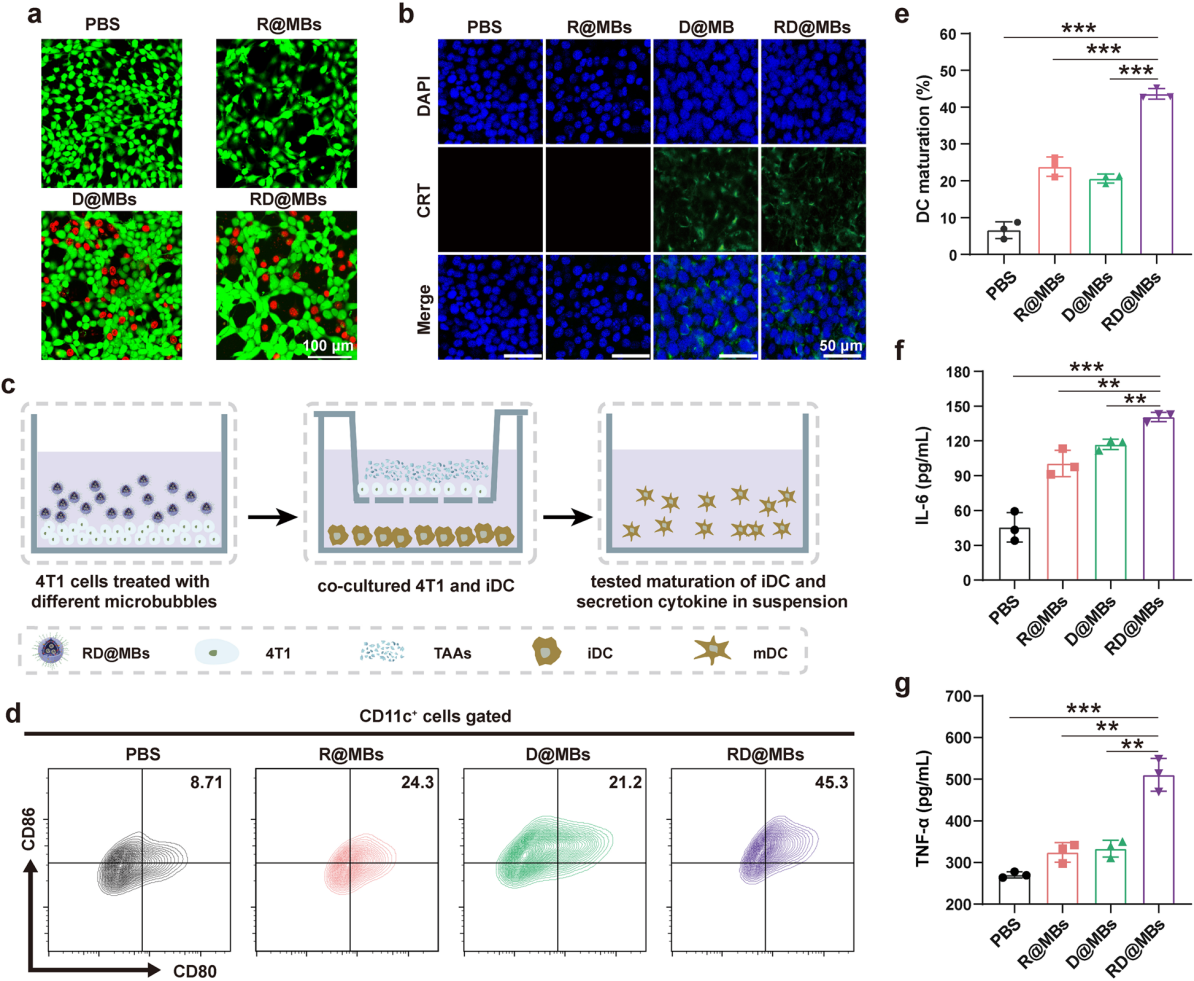
In vitro toxicity of RD@MBs against 4T1 cells and the activation of in vitro immune system. **a** CLSM images of 4T1 cells treated with R@MBs, D@MBs and RD@MBs. 4T1 cells were stained with calcein AM (green, live cells) and propidium iodide (red, dead cells) (scale bar = 25 μm). **b** CLSM images showing the CRT exposure on 4T1 tumor cells after various treatments. Blue, DAPI-labeled nucleus; green, FITC–conjugated anti-CRT antibody-labeled CRT (scale bar = 50 μm). **c** The scheme of the co-culture system. **d**-**e** Representative FCM results of matured DCs (CD11c^+^CD80^+^CD86^+^) after different treatments and corresponding quantitative analysis of DC maturation. **f**-**g** Secretion levels of IL-6 and TNF-α in matured DCs suspensions. Data are expressed as mean ± SD (*n* = 3). Statistical significances were calculated via Student’s t test, *p < 0.05, **p < 0.01 and ***p < 0.001

After successfully demonstrating that DTX treatment can upregulate the expression of PD-L1 on tumor cells and thereby promote the uptake of αPD-L1, which is expected to improve the immunotherapeutic efficacy of PD-L1, we evaluated the ability of the RD@MBs system to activate the immune response in vitro. Dying cells would release TAAs and activate the antitumor immune response. The killing effects of different MBs (R@MBs, D@MBs, RD@MBs) on 4T1 cells at different concentrations were determined by a CCK-8 assay. As shown in Fig. S5, R@MBs had no significant effect on cell viability even at concentrations up to 1000 μg/mL, while DTX-containing MBs showed a gradual decrease in cell viability with increasing MBs concentration, indicating a concentration-dependent cell-killing effect of DTX. In addition, to demonstrate the cytotoxic effect of RD@MBs in vitro, Calcein-AM and PI double staining were employed to distinguish living (green) and dead (red) cells. A certain number of dead cells were observed in the D@MBs-treated and RD@MBs-treated groups (Fig. 2a). In contrast, no red fluorescence was observed in the control and R@MBs groups, indicating that R837 had almost no toxic effect on cells. Multiple studies have shown that extensively damaged cells caused by some chemotherapeutic drugs (*eg.*, paclitaxel, doxorubicin, oxaliplatin, etc*.*) can release damage-associated molecular patterns (DAMPs) to elicit an immune response. This specific cell death modality that triggers an adaptive immune response is called immunogenic cell death (ICD) [29–31].

As a derivative of paclitaxel, DTX might possess similar properties to paclitaxel and can trigger ICD-like effects. CRT, a marker for ICD, acts as an 'eat me' signal for phagocytosis of dying cells to stimulate the antigen-presenting function, which plays a vital role in activating antitumor immunity [32, 33]. After different treatments, 4T1 cells were stained with FITC-conjugated anti-CRT antibody for CLSM observation. As we expected, significant green fluorescence was detected in the DTX-treated groups (D@MBs and RD@MBs) while neglective green could be observed in the control group and R@MBs group (Fig. 2b). These results demonstrated that DTX could induce the release of CRT.

As the most important APCs, DCs play critical roles in innate and adaptive immunity [34]. We have demonstrated that DTX could induce the release of TAAs and CRT, acting as an “eat me” signal for phagocytosis of antigens. After capturing the antigens, immature DCs would migrate to nearby draining lymph nodes, process the antigen into peptides, and undergo maturation. Then, mature DCs would present peptide-major histocompatibility complex (MHC) to naive T cells and finally initiate immune responses. The maturation of DCs is usually accompanied by the upregulation of costimulatory molecules (CD80 and CD86) and the secretion of pro-inflammatory cytokines [35, 36]. A transwell system was employed to investigate the effect of the R837 component in RD@MBs as an immune adjuvant in inducing DC maturation in vitro [37]. Typically, 4T1 cells treated with different MBs were seeded in the upper chamber while BMDC (immature DCs) were seeded in the lower chamber and co-incubated for 24 h (Fig. 2c). Then, cells in the lower chamber were collected for FCM analysis of CD80 and CD86 expression, and the supernatant was collected to detect the secretion of pro-inflammatory cytokines by the corresponding ELISA kits. As shown in Fig. 2d and e, compared with the control group, D@MBs-augmented chemotherapy could slightly help to promote DC maturation (CD80^+^ CD86^+^ CD11c^+^DCs) probably due to the immunity of released TAAs. We found that R837-loaded RD@MBs (43.63 ± 1.81%) could significantly promote DC maturation in comparison with D@MBs (20.6 ± 0.99%). This result could be attributed to the presence of R837 in RD@MBs because R837 alone also promotes DC maturation (23.83 ± 2.15%). Meanwhile, it was found that the concentration of pro-inflammatory cytokines such as IL-6 and TNF-α, which are also indicators of DC activation, remarkably increased (Fig. 2f, g). These results demonstrated that TAAs derived from DTX-treated cells, in combination with R837 as the immune-stimulating adjuvant, efficiently triggered DC maturation.

### In vitro macrophages polarization

Although mature DCs would activate T cells and further induce antitumor immune responses, the immunotherapy efficiency is often suboptimal due to the TIME. As an important component of the TIME, TAMs acted as the first immune defense. TAMs can be mainly divided into two phenotypes: anti-tumoral M1-phenotype (pro-inflammatory) and pro-tumoral M2-phenotype (anti-inflammatory). While the dominating M2-phenotype TAMs secrete immunosuppressive molecules and undermine immunotherapeutic efforts, the M1-phenotype is beneficial for killing cancer cells [7, 38]. MDSCs are a phenotypically heterogeneous cell population, in which immature myelomonocyte precursors (M0-phenotype TAMs) differentiate into TAMs in tumor tissues [39]. M0-phenotype TAMs tend to differentiate towards M2-phenotype due to the abnormal blood vessels and hypoxia in tumor tissues [40]. Of note, TAMs are highly plastic cells and can differentiate into different tumor-related phenotypes. Potential therapeutic strategies targeting TAMs in tumors have been studied and have opened up new avenues for anticancer drug discovery [7, 41]. As DTX and R837 were reported to induce M2 and M0-phenotype TAMs into M1-phenotype, we investigated the polarizing effects of RD@MBs on TAMs.

**Fig. 3:**
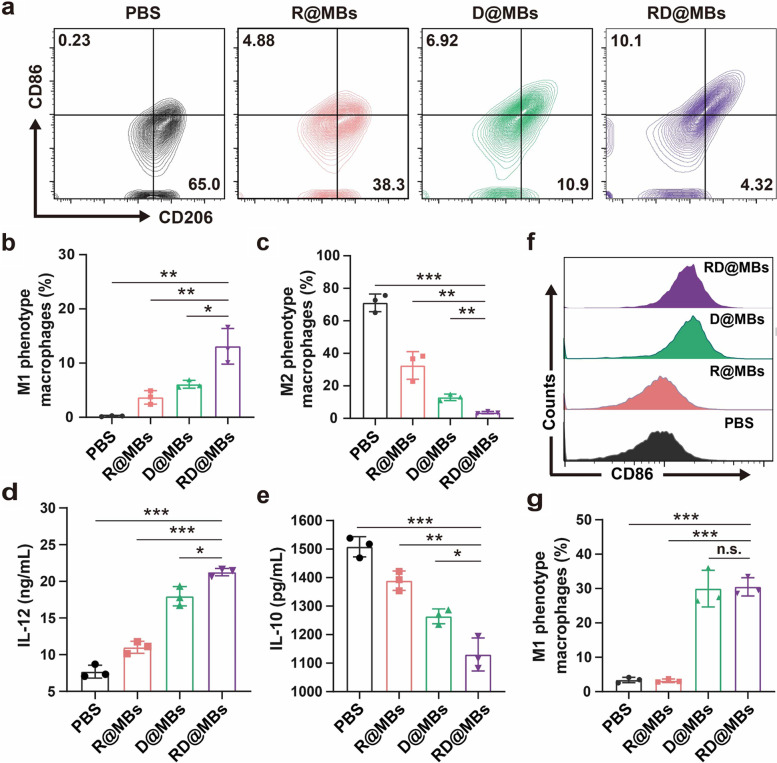
The co-polarization effect of R837 and DTX. **a** FCM results of the expression of M1-phenotype (CD86) and M2-phenotype (CD206) macrophages after different treatments. **b**-**c** Quantifications of M1-phenotype and M2-phenotype TAMs. **d**-**e** Cytokine secretion levels of IL-12 and IL-10 tested by ELISA. **f**-**g** FCM results of M0-to-M1 polarization and quantitative analysis of M1-phenotype macrophages. Data are expressed as mean ± SD (*n* = 3). Statistical significances were calculated via Student’s t test, *p < 0.05, **p < 0.01 and ***p < 0.001

M2-TAMs were generated by pretreating RAW264.7 cells with IL-4 and IL-13. M2-TAMs were treated with different MBs, and M1 and M2-phenotype markers (CD86: M1-TAMs maker; CD206: M2-TAMs maker) were examined by FCM analysis. Compared with the control group, RD@MBs, D@MBs, and R@MBs could greatly decrease the number of M2-TAMs and increase the portion of M1-TAMs, indicating an effective M2-to-M1 transformation (Fig. 3a). In detail, treating M2-TAMs with RD@MBs resulted in the lowest M2-phenotype with a positive rate of 3.55 ± 0.70% and the highest M1-phenotype with a portion of 13.10 ± 3.28% among all groups, which are 12.97 ± 2.00% and 6.09 ± 0.73% in the D@MBs group and 32.57 ± 8.50% and 3.68 ± 1.24% in the R@MBs group, respectively (Fig. 3b, c). The results indicated that the combination of DTX and R837 could synergistically contribute to M2-to-M1 polarization. To further investigate the TAMs polarization, the secretion of IL-10 (M2 macrophage marker) and IL-12 (M1 macrophage marker) in the cell supernatant was analyzed using ELISA kits. As shown in Fig. 3d and e, R@MBs, D@MBs, and RD@MBs could significantly downregulate the section of IL-10 and upregulate the section of IL-12. Moreover, RD@MBs, the combination of DTX and R837 exhibited a synergistic copolarization effect, resulting in a 0.25-fold decrease in IL-10 and 1.76-fold increase in IL-12. These results further demonstrated the capability of the RD@MBs system to polarize M2-phenotype into M1-phenotype TAMs.

It has been reported that DTX can induce MDSCs, which can be considered M0 macrophages in vivo, selectively differentiate into M1-TAMs by inhibiting the STAT3 signaling pathway [23]. We then investigated the effect of RD@MBs on the polarization of M0-to-M1 macrophages. Mouse macrophage line RAW264.7 (M0-TAMs) were treated with different MBs, and cell-surface expression of CD86 was determined by FCM to study the polarization of TAMs. As shown in Fig. 3f and g, CD86 was expressed in only 3.39 ± 0.79% of the untreated M0-TAMs, while 29.97 ± 5.34% and 30.5 ± 2.6% of the DTX-containing D@MBs and RD@MBs group, respectively. The results suggested that DTX can simultaneously induce polarization of M0 towards M1-phenotype. The proportion of CD86-positive cells in the R@MBs group was 3.18 ± 0.52%, which was not significantly different from that in the control group, indicating that R837 could not induce M0-to-M1 polarization. Our results suggested that the DTX not only induced the M2-to-M1 transformation but also promote M0-to-M1 polarization. In addition, R837 also acted as another polarizer to synergize with DTX to further promote the polarization efficiency of TAMs.

### In vivo immune-system activation

**Fig. 4:**
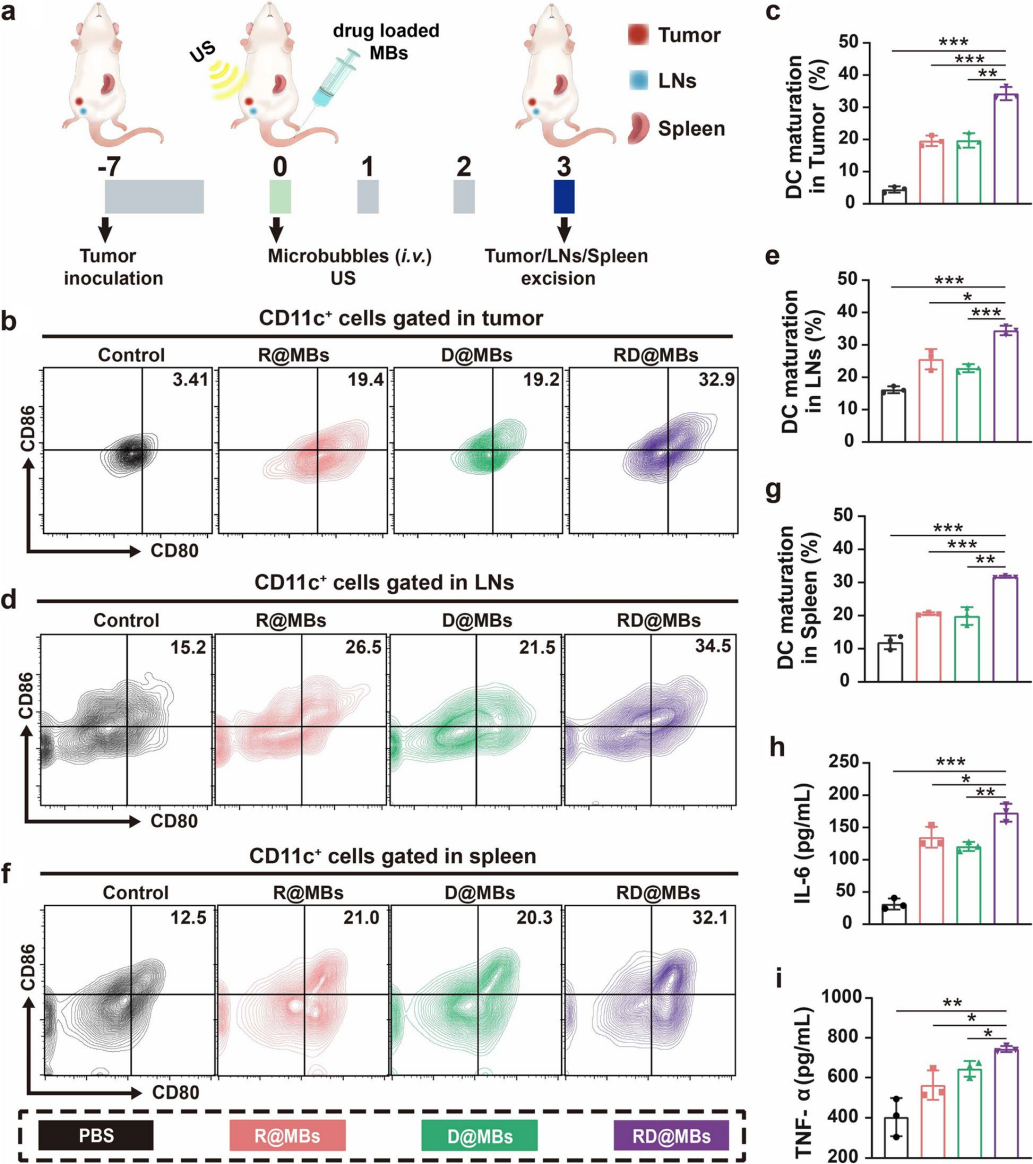
RD@MBs promoted DC maturation and stimulated the secretion of proinflammatory cytokines in vivo. **a** Schematic diagram of the experimental procedure to assess the in vivo DC maturation. **b**-**g** DC maturation (CD11c^+^CD80^+^CD86^+^) in tumor, tumor-draining lymph nodes and spleen. **h**-**i** IL-6 and TNF-α levels in serum, analyzed by ELISA. Data are expressed as mean ± SD (*n* = 3). Statistical significances were calculated via Student’s t test, *p < 0.05, **p < 0.01 and ***p < 0.001

Encouraged by the in vitro results, the in vivo performance of immune response was assessed next. Orthotopic breast tumor-bearing mice were sacrificed on the 3^rd^ day after different treatments, and tumors, lymph nodes, and spleen were excised for FCM analysis of DC maturation (Fig. 4a). RD@MBs induced high levels of DC maturation in tumors, lymph nodes, and spleen (Fig. 4b-g), which were 6.67-fold, 1.13-fold, and 1.66-fold, respectively, in comparison to the control. Cytokine secretion is also a typical indication of immune responses; thus, we further assessed the serum levels of IL-6 and TNF-α. Consistent with the DC maturation results, the combination of R837 and DTX triggered the highest levels of immune cytokines, including IL-6 (Fig. 4h) and TNF-α (Fig. 4i), again demonstrating that the R837 can assist in enhancing the immune response. Therefore, TAAs and CRT derived from DTX-induced cell debris, combined with R837 as an immunostimulatory adjuvant, can effectively trigger DC maturation. Although intravenous injection of immune adjuvants was known to cause serious side effects such as cytokine storm, there were no unscheduled deaths throughout the study, indicating that the delivery of R837 by UTMD technology and had high biological safety.

### RD@MBs plus αPD-L1 blockade for suppression of tumors

Tumor metastasis is one of the leading causes of cancer-associated mortalities [42], and it is usually accompanied by resistance to conventional treatments. Therefore, the desirable cancer therapy should not only eradicate the primary tumor but also recognize, suppress, and remove any residual tumor tissue at metastatic sites. PD-1/PD-L1 checkpoint blockade has been shown to promote anti-tumor immunity by reducing CTLs depletion, especially when combined with other treatment regimens, the anti-tumor effect can be significantly improved [43]. The combination of ICB with chemotherapy has attained a synergistic effect on combating cancer by enhancing tumor immunogenicity and increasing T cell infiltration [44]. Based on the aforementioned results, the therapeutic efficacy of RD@MBs in combination with αPD-L1 against primary tumors and artificial mimic of metastasis.

**Fig. 5:**
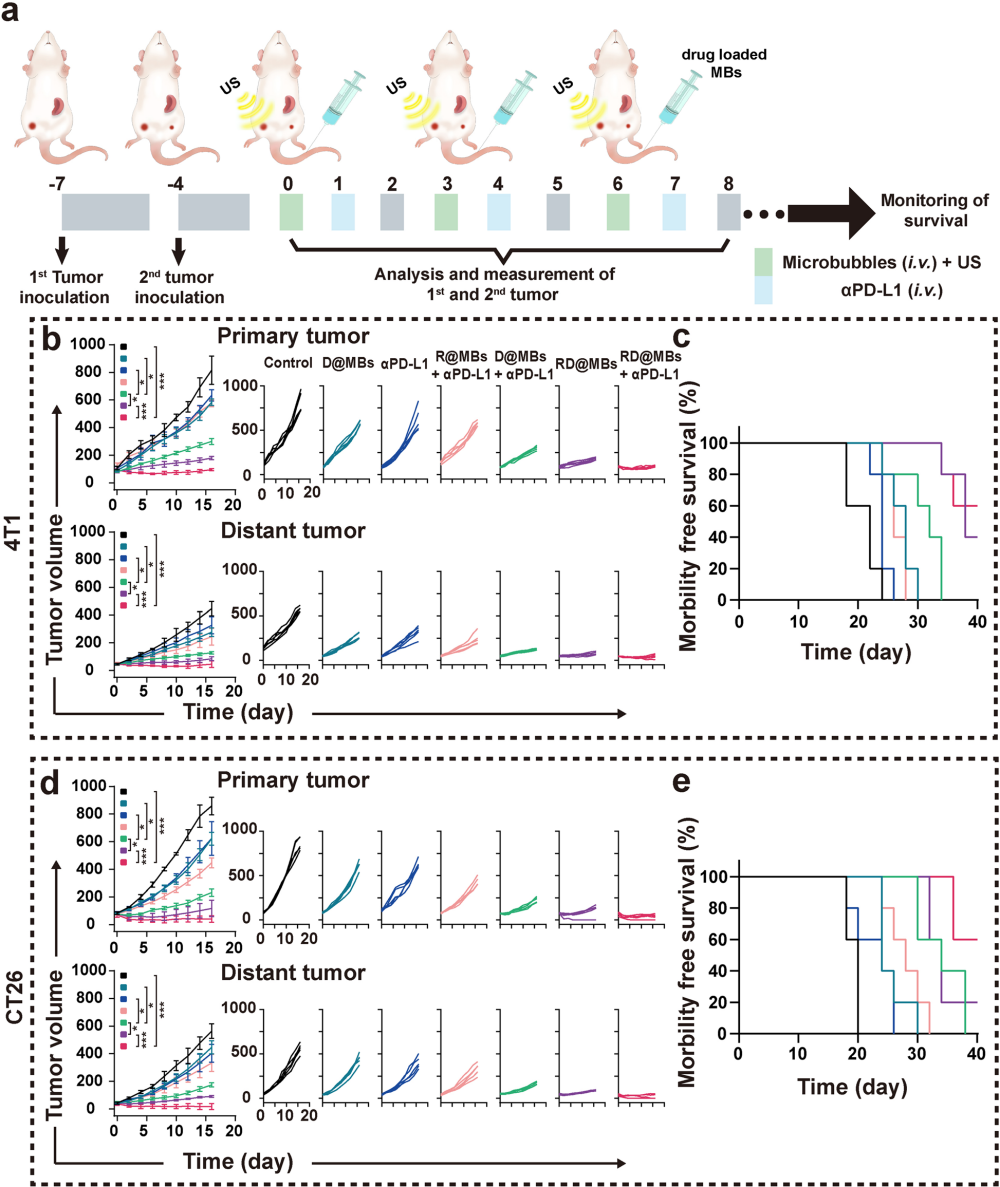
In vivo antitumor and metastasis of RD@MBs + αPD-L1 in murine 4T1 and colorectal cancer models. **a** Schematic illustration of the experimental schedule on tumor bearing mice. **b** Average and individual tumor growth curves of 4T1 orthotopic tumor bearing mice primary and distant tumor after different treatments. **c** Survival rate of mice after different treatment on 4T1 orthotopic tumor bearing mice. **d** Average and individual tumor growth curves of CT26 subcutaneous tumor bearing mice primary and distant tumor after different treatments. **e** Survival rate of mice after different treatments on CT26 subcutaneous tumor bearing mice. Data are expressed as mean ± SD (*n* = 5). Statistical significances were calculated via Student’s t test, *p < 0.05, **p < 0.01 and ***p < 0.001

The procedure is illustrated in Fig. 5a. After the first orthotopic breast cancer was inoculated on day -7, the same amount of 4T1 cells was inoculated into the contralateral mammary fat pad to simulate distant metastasis on day -4. At day 0, mice were randomly divided into seven groups (*n* = 5 per group): (1) Control, (2) D@MBs, (3) αPD-L1, (4) R@MBs + αPD-L1, (5) D@MBs + αPD-L1, (6) RD@MBs and (7) RD@MBs + αPD-L1. The primary tumors were treated with US irradiation immediately after each intravenous injection of different MBs. αPD-L1 (30 μg per mouse) was intravenously administrated the next day. The same treatments were also performed on the 3^rd^ and 6^th^ days. Tumor volumes and body weights were monitored every 2 days until the tumor volume reached 1000 mm^3^ (the mice were euthanized when the tumor volume exceeded 1000 mm^3^).

The therapeutic outcomes of the different groups for primary and mimic distant tumors are summarized in Fig. 5b and Fig. S6a, b. The tumor volume growth curves indicate that D@MBs, αPD-L1, R@MBs + αPD-L1, D@MBs + αPD-L1, RD@MBs, and RD@MBs + αPD-L1 show tumor-suppressive outcomes. Compared with the 16-day tumor volume of the control group, D@MBs、αPD-L1、R@MBs + αPD-L1 exhibited a mild antitumor effect; the tumor growth inhibition (TGI) of the primary/ metastatic tumors was 30–40%. Compared with D@MBs, RD@MBs group containing the R837 shows a better tumor inhibition effect, indicating the importance of immune adjuvant R837 in immune response after chemotherapy. D@MBs + αPD-L1 showed a better tumor growth inhibitory effect as compared to D@MBs, which could be ascribed to the DTX-triggered ICD-like responses enhanced tumor immunogenicity, and further potentiated the immunotherapeutic effect of αPD-L1.

As expected, the best tumor inhibition effect is achieved from the RD@MBs + αPD-L1 group, and the tumor inhibition rate of primary and distant tumors reached 88.28 ± 1.04% and 89.86 ± 5.76% in the combination of DTX, R837, and αPD-L1. The survival curves (Fig. 5c) showed that the mice in the monotherapy group (D@MBs, αPD-L1) all experienced lethality within 24–30 days, and RD@MBs, the combination DTX and R837, significantly prolonged the survival time (≤ 40 days) as compared to the monotherapy, while 3 mice in the RD@MBs + αPD-L1 survived ≥ 40 days. To assess metastatic dissemination, lung tissues, the most common site of metastasis, were collected and fixed with Bouin when the mice became moribund, and quantification of lung surface metastases was determined. The gross appearance of lung nodules revealed that the RD@MBs + αPD-L1 group exerted excellent performance in inhibiting lung metastasis than the other groups (Fig. S6c). The quantitative results also confirmed that the RD@MBs + αPD-L1 synergistic therapeutic strategy substantially reduced lung nodules as compared to the control, *i.e.*, 45 ± 7.97 V.S. 13.4 ± 4.88 (Fig. S6d). Although, the TGI of the RD@MBs + αPD-L1 treatment is already approximately 10% higher than that of RD@MBs in primary/ metastatic 4T1 tumors. The difference is even more significant in terms of the lung metastatic inhibition, where the number of pulmonary metastatic nodules in RD@MBs + αPD-L1 group was only half of that in RD@MBs group.

In addition, to evaluate the therapeutic effect, hematoxylin and eosin (H&E) staining and terminal deoxynucleotidyl transferase dUTP nick end labeling (TUNEL) staining were performed on the primary tumor to observe the necrosis and apoptosis, and the proliferation of the metastatic tumor was assessed by PCNA staining (Fig. S6e). H&E staining shows that RD@MBs + αPD-L1 resulted in the largest area of nuclear atrophy and fragmented cell necrosis in the primary tumor among all groups. The TUNEL assays show that RD@MBs + αPD-L1 induced the greatest degree of apoptosis. And the PCNA staining revealed that RD@MBs + αPD-L1 also induced the maximal inhibition of metastatic tumor proliferation. These results further suggest the potent antitumor immune effects of RD@MBs + αPD-L1 synergistic therapy. RD@MBs combined with αPD-L1 remodeled the TIME, transformed immune "cold" tumors into "hot", and effectively suppressed primary and distant metastatic tumors. Furthermore, there is no significant difference in body weights among groups during the period of treatment, indicating that this synergistic therapeutic strategy is of good biocompatibility (Fig. S6f).

Based on the aforementioned results, RD@MBs + αPD-L1 could elicit strong and effective antitumor immune responses in the 4T1 breast cancer orthotopic models. We envision that this synergistic chemoimmunotherapy strategy could potentially be used in other tumor types. To further explore the therapeutic efficacy, the bilateral subcutaneous CT26 model was established and received the same treatment as the aforementioned protocol. As shown in Fig. 5d, e and Fig. S6g, h, the in vivo therapeutic results were similar to the 4T1 orthotopic breast cancer model. The immune response elicited after primary tumor chemotherapy based on RD@MBs can effectively inhibit distant metastasis and prolong the survival of mice. No significant body weight loss of mice in all groups was found throughout the therapeutic period (Fig. S6i), once again substantiating the biocompatibility and biosafety. This excellent therapeutic efficacy in different tumor types demonstrates that this new chemoimmunotherapy strategy can be extended to various other types of tumors.

### Mechanism of antitumor immune responses

**Fig. 6:**
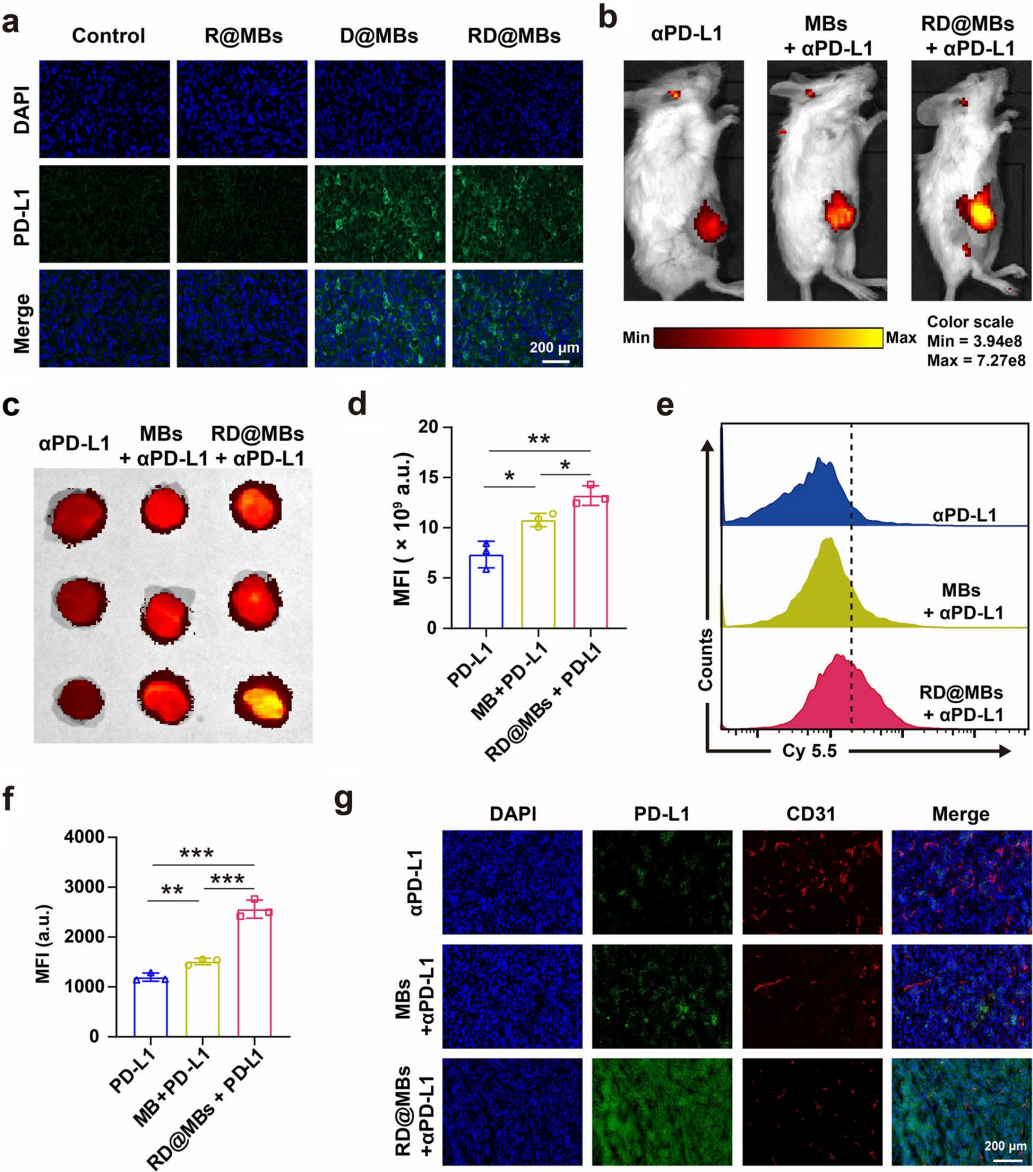
Increased PD-L1 expression and cellular uptake in tumor tissues after DTX stimulation. **a** Immunofluorescence staining images for PD-L1 expression of 4T1 tumor sections after various treatments. Blue, DAPI-labeled nucleus; green, FITC–conjugated anti-PD-L1 antibody-labeled PD-L1 (scale bar = 50 μm). **b** In vivo fluorescence images of 4T1 tumor dissected from mice after different treatments (αPD-L1, MBs + αPD-L1, RD@MBs + αPD-L1). **c** Ex vivo fluorescence images of 4T1 tumor dissected from mice after different treatments (αPD-L1, MBs + αPD-L1, RD@MBs + αPD-L1). **d** Corresponding MFI of ex vivo fluorescence images (*n* = 3). **e** FCM results of uptake of Cy5.5-labeled αPD-L1 on 4T1 tumor organ after different treatments. **f** Corresponding quantification of uptake of Cy5.5-labeled αPD-L1 (*n* = 3). **g** Frozen section images of distribution of Cy5.5-labeled αPD-L1 on 4T1 tumor after different treatments. Blue, DAPI-labeled nucleus; green, Cy5.5-labeled αPD-L1; red, anti-CD31 antibody labeled blood vessels (scale bar = 200 μm). Data are expressed as mean ± SD. Statistical significances were calculated via Student’s t test, *p < 0.05, **p < 0.01 and ***p < 0.001

The chemoimmunotherapy strategy of RD@MBs in combination with αPD-L1 achieved satisfactory therapeutic effects in different tumor models. We further investigated the underlying mechanism of the therapeutic strategy. The accumulation of αPD-L1 in tumor site and tumor-infiltrating T lymphocytes are both key parameters in tumor immunotherapy. First, the effect of RD@MBs on PD-L1 expression and αPD-L1 uptake in the tumor were investigated in orthotopic murine models of breast cancers. Immunofluorescence staining in tumor tissues for PD-L1 of different treatment groups 24 h post US irradiation. As shown in Fig. 6a, also consistent with the results of in vitro cell experiments, obvious green fluorescence was observed in the D@MBs group and the RD@MBs group, indicating that DTX upregulated the expression of PD-L1 in tumor cells. Next, we further investigated the uptake of αPD-L1 in vivo. Cy5.5-labeled αPD-L1 was intravenously injected into the tumor-bearing mice, and at 24 h post-injection, in vivo fluorescence imaging was carried out on mice and the tumor tissues were dissected for ex vivo fluorescence imaging and FCM analysis. As shown in Fig. 6b to f, the fluorescence intensity of the RD@MBs-treated group was higher than that of the control and the MBs-treated group in both *in/*ex vivo fluorescence imaging and FCM analysis. Interestingly, the MFI in the MBs-treated group without drug loading is higher than the control, which may be attributed to the cavitation effect mediated by the UTMD technique. It has been extensively reported that the UTMD technique is employed to enhance the permeability of tumor tissues for better accumulation and penetration of therapeutic drugs [45]. The tumor tissues were frozen-sectioned to observe the distribution of αPD-L1. In the control group, only weak Cy5.5 (Green) fluorescence was observed and mainly distributed around tumor blood vessels stained by anti-CD31 (red), while more dense and stronger green fluorescence even distant from tumor vessels was observed in RD@MBs group (Fig. 6g). The high uptake and desirable penetration of αPD-L1 could be ascribed to DTX-induced upregulation of PD-L1 expression in tumor cells and the cavitation effect mediated by UTMD technology. In addition, the synergistic regulation of the tumor microenvironment by R837 and DTX may also contribute to the enhanced retention and penetration of αPD-L1 in tumors.

**Fig. 7:**
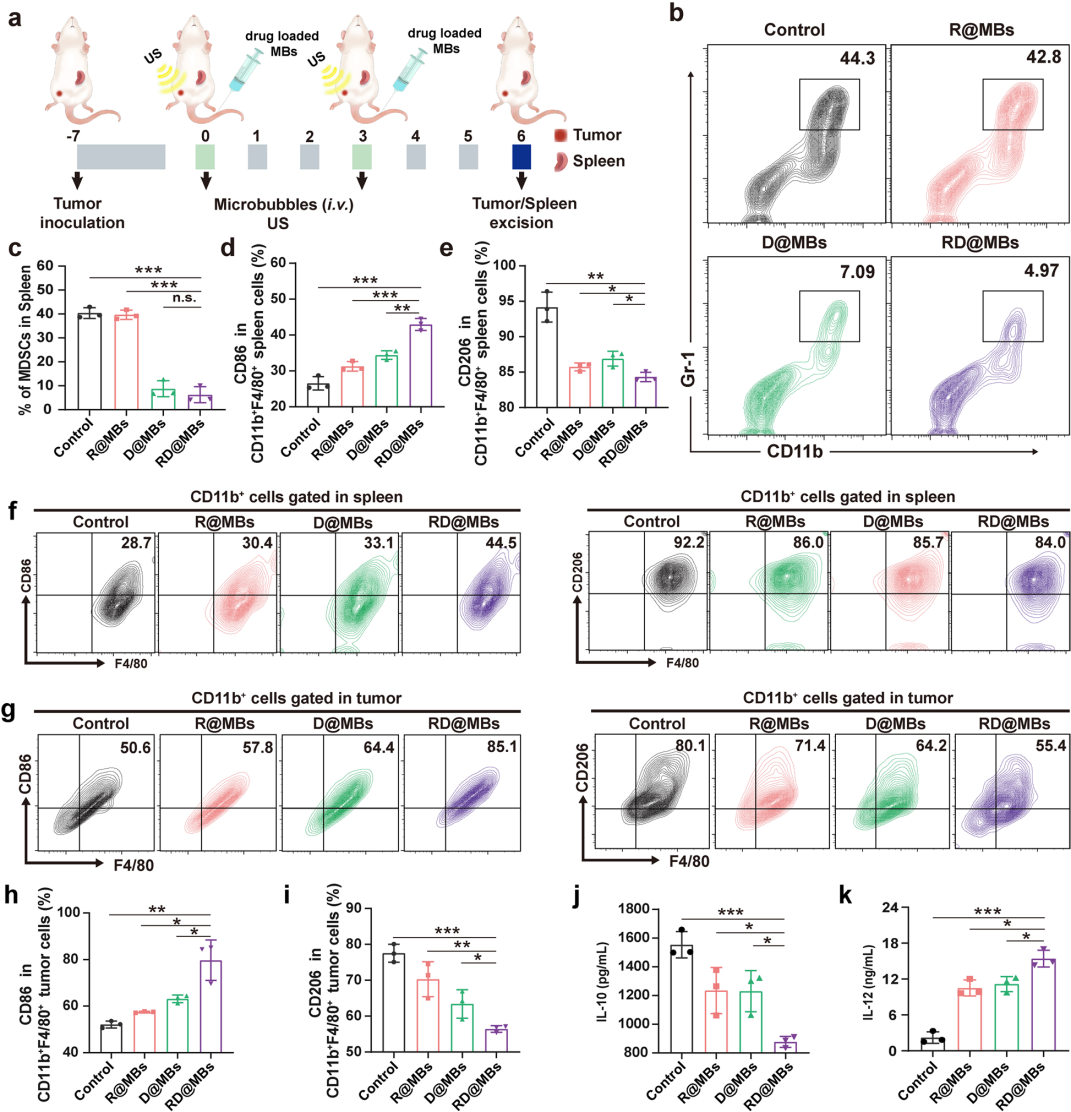
In vivo polarization of TAMs. **a** Schematic illustration of the experiment design for in vivo FCM. **b**-**c** FCM and corresponding quantification of MDSCs (CD11b^+^Gr-1^+^) in spleens. **d**-**e** The proportions of M1-TAMs (CD11b^+^F4/80^+^CD86^+^) and M2-TAMs (CD11b^+^F4/80^+^CD206^+^) in spleens. **f** FCM results of M1-TAMs (CD11b^+^F4/80^+^CD86^+^) and M2-TAMs (CD11b^+^F4/80^+^CD206^+^) in spleens. **g** FCM results of M1-TAMs (CD11b^+^F4/80^+^CD86^+^) and M2-TAMs (CD11b^+^F4/80^+^CD206^+^) in tumors. **h**-**i** The proportions of M1-TAMs (CD11b^+^F4/80^+^CD86^+^) and M2-TAMs (CD11b^+^F4/80^+^CD206^+^) in tumors. **j**-**k** Quantification of IL-10 and IL-12 levels in serum. Data are expressed as mean ± SD (*n* = 3). Statistical significances were calculated via Student’s t test, *p < 0.05, **p < 0.01 and ***p < 0.001

Driven by the promising in vitro polarization result, we investigated whether RD@MBs could effectively induce the polarization of macrophages in vivo to alleviate the immunosuppressive microenvironment of tumors. The mice were sacrificed on the 6^th^ day after different treatments, the polarization of the TAM phenotype and infiltration of MDSCs were analyzed by FCM (Fig. 7a). RD@MBs significantly reduced the percentage of immunosuppressive MDSCs and M2-TAMs and increased M1-TAMs in the spleen. In detail, MDSCs (CD11b^+^Gr-1^+^) in the control group and R@MBs-treated group were 40.41 ± 1.85% and 39.71 ± 1.29%, respectively, while MDSCs in the D@MBs and RD@MBs decreased to 8.79 ± 2.77% and 6.28 ± 2.71% (Fig. 7b, c). M2-TAMs (CD11b^+^F4/80^+^CD206^+^) in RD@MBs decreased from 94.17 ± 1.72% to 84.33 ± 0.54%, and M1-TAMs (CD11b^+^F4/80^+^CD86^+^) increased from 26.53 ± 1.54% to 42.93 ± 1.35% correspondingly (Fig. 7d - f). Accordingly, M2-TAMs decreased from 77.53 ± 2.50% to 56.43 ± 0.91% and M1-TAMs increased from 52.13 ± 1.50% to 79.73 ± 8.70% in tumor tissues (Fig. 7g – i). Consistent with the in vitro results, the combination of R837 and DTX exhibited satisfactory copolarization effects in both spleen and tumor tissues. In addition, the levels of serum macrophage-polarizing cytokines from the treated mice were determined by ELISA assays. As shown in Fig. 7j and k, RD@MBs significantly reduced the secretion of IL-10 and increased IL-12, indicating that the combined treatment of R837 and DTX effectively induced the polarization of macrophages in the tumor microenvironment and evidently activated the antitumor inflammatory immune response.

Furthermore, the immunofluorescence assay of tumor tissues was measured to demonstrate the expression of the M2-phenotype marker (CD206, VEGF, MMP-9), and M1-phenotype marker (CD86). As shown in Fig. S7, compared with the control group, RD@MBs significantly upregulated the expression of CD86 (green fluorescence) and downregulated the expression of CD206 (red fluorescence), which further demonstrated the role of RD@MBs in polarizing TAMs into an M1-phenotype. In the meantime, RD@MBs attenuated the expression of VEGF and MMP-9 (Fig. S8). Studies have documented that VEGF and MMP-9 are key mediators of abnormal angiogenesis and can regulate most of the steps in angiogenesis [5, 46]. The decrease of VEGF and MMP-9 can promote the normalization of tumor blood vessels, restore the normal function of vascular endothelial, and facilitate the transport, adhesion, and infiltration of T cells to tumor tissues. Overall, DTX combined with R837 remodeled suppressive M2-TAMs into pro-inflammatory M1-TAMs and reduced MDSCs, transforming immunosuppressive "cold" tumors into highly immunogenic "hot" tumors, which would increase tumor sensitivity to immune responses and lay the foundation for efficient tumor chemoimmunotherapy.

**Fig. 8:**
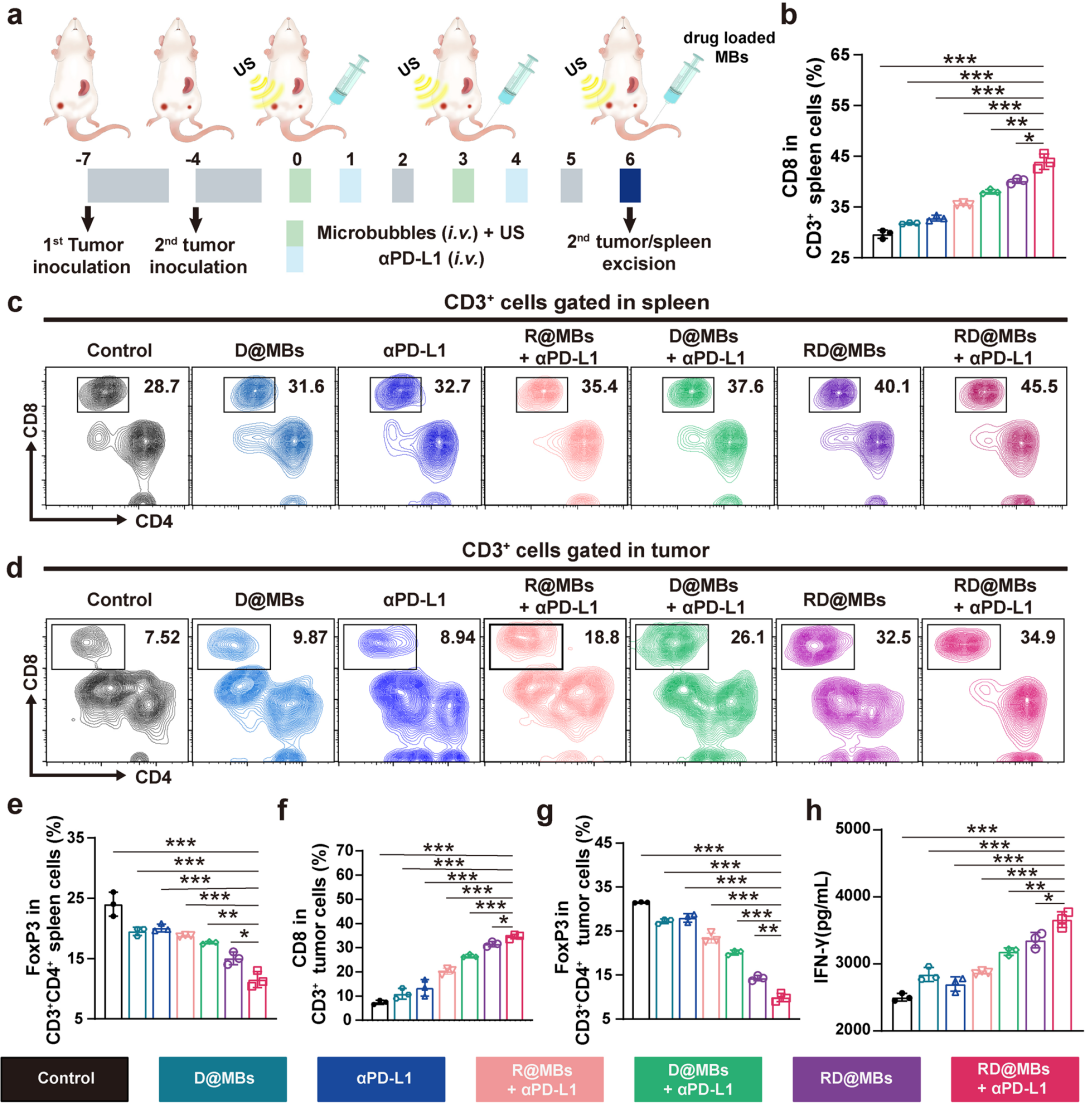
Combination of RD@MBs with αPD-L1 chemoimmunotherapy activated systematic antitumor response. **a** Schematic illustration of the experiment design for antitumor immune responses. **b** The proportions of CTLs (CD3^+^CD4^−^CD8^+^) in spleens. **c** FCM results of CTLs (CD3^+^CD4^−^CD8^+^) in spleen. **d** FCM results of CTLs (CD3^+^CD4^−^CD8^+^) in tumor. **e** The proportions of Tregs (CD3^+^CD4^+^Foxp3^+^) in spleens. **f**-**g** The proportions of CTLs (CD3^+^CD4^−^CD8^+^) and Tregs (CD3^+^CD4^+^Foxp3^+^) in tumors. **h** IFN-γ levels in serum. Data are expressed as mean ± SD (*n* = 3). Statistical significances were calculated via Student’s t test, *p < 0.05, **p < 0.01 and ***p < 0.001

T lymphocytes play an important role in antitumor immunity. Next, T lymphocytes in tumors and spleens were assessed using 4T1 bilateral tumor models (Fig. 8a). Among tumor-infiltrating T lymphocytes, CD8^+^ T cells, which are also called CTLs, are the predominant pillar that was responsible for the strong antitumor immune responses [47], while CD4^+^ T cells act as helper T cells, and can be divided into two types according to the level of Foxp3 markers, namely effective T cells (Teff, CD4^+^Foxp3^−^) and regulatory T cells (Tregs, CD4^+^Foxp3^+^) [48]. Tregs are a distinct lineage of CD4^+^ T lymphocytes that regulate immune suppression and maintain self-tolerance. Therefore, the proportion of CTLs (CD3^+^CD4^−^CD8^+^) and Treg (CD3^+^CD4^+^Foxp3^+^) in metastases and spleen was analyzed. As shown in Fig. 8b-g and Fig. S9a, b, a small number of CTLs and a large number of Tregs were observed in the control group, which could be attributed to innate immune resistance. Only a small increase in CTLs infiltration and a decrease in Treg frequency were observed in the pure αPD-L1 treated group, indicating that αPD-L1 only cannot exert a good therapeutic effect due to the influence of the TIME. In the RD@MBs group, the infiltration of CTLs was significantly increased, and the frequency of Treg was also significantly decreased, indicating that the copolarizing effect of TAMs induced synergistically by R837 and DTX at the primary tumor site greatly enhanced CTLs activation, proliferation, and migration, as well as the suppression on Treg cells. Notably, RD@MBs + αPD-L1 elicited the highest degree of CTLs infiltration (a 3.65-fold and 0.48-fold increase of CTLs) and reduction in Treg cells (a 2.82-fold and 1.08-fold decrease of Treg) both in distant metastases and in the spleen, respectively, compared with the control group. These results suggest that the relief of the TIME by RD@MBs significantly promotes the infiltration of CTLs at tumor sites and improves the efficacy of αPD-L1.

The infiltration of CTLs in primary tumors was also observed by immunofluorescence staining. As shown in Fig. S10, in the tissue sections of the D@MBs and αPD-L1 groups, the green fluorescence of CD8^+^ T cells was relatively weak, while the much stronger green was observed in the D@MBs + αPD-L1 group. This may be attributed to the upregulation of PD-L1 expression by DTX, which increases the uptake of αPD-L1, and the regulation of TIME, thereby significantly increasing the infiltration in tumors. Comparatively, when R837, DTX, and αPD-L1 act in synergy, the RD@MBs + αPD-L1 group showed the strongest green fluorescence, indicating the most abundant infiltrating CTLs. CTLs could release interferon-γ (IFN-γ) to promote the immune microenvironment and kill tumor cells [4], therefore the content of IFN-γ in mouse serum was determined by ELISA. Consistent with the results of FCM and immunofluorescence staining, the highest level of IFN-γ was observed in the combination treatment group (Fig. 8h). The inhibition of primary tumor and distant metastasis by RD@MBs + αPD-L1 may be attributed to the upregulation of PD-L1 expression on tumor cells with DTX, which promotes the uptake of PD-L1. Meanwhile DTX and R837 multiply alleviated TIME (TAMs, MDSCs, Treg) and promoted the infiltration of T cells into tumors.

## Discussion

Recent progress in basic research and clinical practice of PD-1/PD-L1 ICB therapy has improved the survival rate of cancer patients. However, the stumbling block of low response rate still needs to be further solved. The infiltration of T cells and the accumulation of αPD-L1 in tumor sites are critical for PD-L1-mediated ICB therapy response rate. In this study, we described a versatile UTMD drug delivery system integrated with chemotherapy and immunotherapy. This drug delivery system has effectively improved the response rate of PD-L1 ICB therapy through the following aspects. (1) DTX-based chemotherapy releases TAAs and initiates the activation, proliferation, and recruitment of T cells. Assisted by R837, the immune responses are significantly enhanced. (2) DTX and R837 effectively reduced immunosuppressive M2-TAMs and MDSCs, and alleviated immunosuppression. (3) DTX upregulates the expression of PD-L1 on tumor cells, which can promote the uptake of αPD-L1 and further improve the response rate of ICB. Due to the large amount of PD-L1 expression in spleen and LNs, the enrichment of αPD-L1 in tumors after intravenous injection was not satisfied, so a generous portion of the free aPD-L1 may bind to normal tissues provokes on-target off-tumor immune-related adverse effects (irAEs) [49]. Increasing PD-L1 expression on tumor cells may reduce the occurrence of adverse reactions. (4) ICB effectively depletes Treg and improves the activities of CTLs in tumor tissues. (5) UTMD technique promotes the accumulation and penetration of αPD-L1 in tumor tissues, which plays an important role in improving the efficacy of ICB.

With a glimpse at the recent advances of ICB, the combination paradigms between traditional cancer treatment modalities (*e.g.*, radiotherapy, photodynamic therapy, photothermal therapy and sonodynamic therapy (SDT)) and PD-1/PD-L1 or CTLA-4 can improve the effects of cancer immunotherapy. Compared with recent combined treatment strategies, this established strategy of UTMD technique mediated chemoimmunotherapy offers key advantages. First, in comparison with radiotherapy, photodynamic therapy and photothermal therapy, ultrasonic irradiation is a noninvasive and safe treatment modality with higher tissue penetration depth, and can be widely used in many tumor models. Second, US-triggered sonodynamic therapy, as a non-invasive therapeutic modality, has widely reported in combination with ICB for cancer immunotherapy. In a typical SDT process, US activates the sonosensitizers to generate cytotoxic reactive oxygen species (ROS). However, some inherent characteristic feature of the tumor microenvironment, some intrinsic characteristics of the tumor microenvironment, such as hypoxia and high glutathione (GSH) [50], severely diminish the therapeutic effects of SDT and cannot effectively induce immunogenic cell death (ICD) to activate anti-tumor immune response. Meanwhile, oxygen consumption during SDT will further aggravate tumor hypoxia, reduce the efficiency of SDT and promote tumor metastasis [51]. UTMD technology proposed in this study are exempt from adverse hypoxia and high GSH in the tumor microenvironment. The potential toxicity of sonosensitizers also hampers clinical translation of SDT [4]. Reagents used in this combinatorial therapy, such as DTX, R837, αPD-L1, without any extra modifications, are all U.S. Food and Drug Administration-approved agents. Furthermore, various TAMs polarization strategies have been reported, but most are limited to inducing M2-to-M1 transformation. In this study, DTX also induced the polarization of M0-to-M1 [48]. Therefore, RD@MBs possess enormous potential in alleviating the immunosuppressive microenvironment and may greatly improve the efficiency of immunotherapy. While there have been numerous cases of chemotherapeutic drugs delivered by MBs for tumor therapy, the delivery of immune adjuvants by MBs is rarely reported. Moreover, compared with other drug delivery systems (*e.g.*, liposomes, mesoporous, and metal–organic framework), UTMD technique holds great potential in efficient and localized safe drug delivery due to its safety and non-invasive characteristics [52]. UTMD technique can achieve the on-demand release of DTX and R837 at the tumor site, which greatly reduces the side effects of chemotherapy drugs and immune adjuvants. Thus, this drug-loaded MBs delivery system-based chemoimmunotherapy is easy to practice in the clinic.

## Conclusion

In this work, we rationally designed a UTMD drug delivery system for DTX/R837-enhanced immunotherapy against cancer, and successfully improved the efficacy of ICB by remodeling the TIME and promoting intertumoral accumulation of αPD-L1. We demonstrated a highly efficient chemoimmunotherapy against tumors. In this system, a chemotherapeutic drug DTX and immune adjuvant R837 were loaded into MBs (RD@MBs). Upon noninvasive US irradiation, DTX can be released and induce tumor cell death. The dying tumor cells would release TAAs and T-cell stimulating factors (CRT, CD80, CD86), activating antitumor immune response. Also, DTX exhibited strong immune regulation capabilities, such as upregulating the expression of PD-L1 on tumor cells, promoting the uptake of αPD-L1, inducing the polarization of M2-phenotype TAMs to M1-phenotype, and reducing the proportion of MDSCs. Together with TAAs, R837 promoted DCs maturation and cytokine secretion. Furthermore, R837 could act as a regulator of TAMs polarization, which could synergistically alleviate TIME with DTX, and promote the activation, proliferation and recruitment of T cells to tumor tissues, reducing the resistance of ICB therapy and improving the response rate. In addition, UTMD technique promoted the delivery efficacy of αPD-L1, which was beneficial to ICB therapy. We demonstrated that, using two murine tumor models (*i.e.*, orthotopic xenograft 4T1 breast cancer mouse model and bilateral subcutaneous CT26 model), that RD@MBs + αPD-L1 synergistic therapy not only effectively inhibited the growth of primary tumors, but also significantly inhibited the mimic distant tumors as well as lung metastases. Considering its biocompatibility, biosafety, and efficiency, chemoimmunotherapy based on RD@MBs + αPD-L1 can be a promising therapeutic modality against cancer.

## Supplementary Information

**Additional file 1:** **Supporting Figure S1.** Characterization of RD@MBs. (a) Light microscopy image of RD@MBs (scale bar = 10 μm). (b-c) The standard curves of DTX and R837 measured by LC-MS. **Supporting Figure S2.** Biosafety of RD@MBs. (a) Cell viabilities of 4T1 cells treated with different concentrations of RD@MBs (without US irradiation) (*n* = 3). (b) Heatmap of the blood biochemical and blood routine analysis (*n* = 5). (c) H&E images of major organs (heart, liver, spleen, lung and kidney) treated with different concentrations of RD@MBs for *in vivo* biosafety evaluation. Data are expressed as mean ± SD. **Supporting Figure S3.** UTMD drug delivery system. (a-b) In vitro US imaging and the corresponding echo intensities of RD@MBs at various concentrations (20, 40, 60, 80, 100 µg/mL) (*n* = 3). (c-d) *In vivo* US imaging and corresponding echo intensities of tumor regions at different time points (*n* = 3). **Supporting Figure S4. **UTMD drug delivery system. (a-b) FCM results of drug release in tumor sites and the corresponding quantitative analysis of MFI. **Supporting Figure S5.** Cell viabilities of 4T1 cells treated with different concentrations of R@MBs, D@MBs and RD@MBs (*n* = 3). Data are expressed as mean ± SD. Statistical significances were calculated *via* Student’s t test, *p < 0.05, **p < 0.01 and ***p < 0.001. **Supporting Figure S6.** (a-b) Primary and distant tumor TGI after different treatments in 4T1 orthotopic tumor bearing mice (*n* = 5). (c-d) Representative digital photos of tumor nodules in the 4T1 orthotopic tumor bearing mice lungs and corresponding quantification of the numbers of lung nodules (*n* = 5). (e) H&E and TUNEL staining images of primary tumor in 4T1 orthotopic tumor bearing mice. PCNA staining images of distant tumor in 4T1 orthotopic tumor bearing mice. (Scale bar = 200 μm). (f) Body weight of the 4T1 orthotopic tumor bearing mice after different treatments (*n* = 5). (g-h) Primary and distant tumor TGI after different treatments in CT26 subcutaneous tumor bearing mice (*n* = 5). (i) Body weight of the CT26 subcutaneous tumor bearing mice after different treatments (*n* = 5). Data are expressed as mean ± SD. Statistical significances were calculated *via* Student’s t test, *p < 0.05, **p < 0.01 and ***p < 0.001. **Supporting Figure S7.** Immunofluorescence staining images for expression of CD86 and CD206 in 4T1 tumor tissues after different treatments. Blue, DAPI-labeled nucleus; green, anti-CD86 antibody-labelled M1-TAMs; red, anti-CD206 antibody-labeled M2-TAMs. (Scale bar = 200 μm). **Supporting Figure S8.** Immunofluorescence staining images for expression of MMP-9 and VEGF in 4T1 tumor tissues after different treatments. Blue, DAPI-labeled nucleus; green, anti-MMP-9 antibody-labelled MMP-9; red, anti-VEGF antibody-labeled VEGF (scale bar = 200 μm).**Supporting Figure S9.** (a) FCM results of Tregs (CD3^+^CD4^+^Foxp3^+^) in spleen. (b) FCM results of Tregs (CD3^+^CD4^+^Foxp3^+^) in tumor. **Supporting Figure S10.** Immunofluorescence staining images for infiltration of CTLs in primary tumor of 4T1 orthotopic tumor bearing mice after different treatments. Blue, DAPI-labeled nucleus; green, anti-CD8 antibody-labelled CTLs (scale bar = 100 μm).

## Data Availability

Additional data related to this study may be requested from the corresponding author upon reasonable request without breaching participant confidentiality.

## Abbreviations

DTX: Docetaxel
R837: Imiquimod
RD@MBs: DTX/R837 coloaded microbubbles
αPD-L1: Anti-Programmed Cell Death-Ligand 1 antibody
ICB: Immune checkpoint blockade
UTMD: Ultrasound-targeted microbubble destruction
TAMs: Tumor-associated macrophages
Treg: Regulatory T cells
MDSCs: Myeloid-derived suppressor cells
TAAs: Tumor-associated antigens
APC: Antigen-presenting cell
TME: Tumor microenvironment
ICD: Immunogenic cell death
DCs: Dendritic cells
VEGF: Vascular endothelial growth factor
MMP9: Matrix metalloproteinase-9
CCK-8: Cell counting kit-8
CLSM: Confocal laser scanning microscope
FCM: Flow cytometry
HE: Hematoxylin & eosin
TUNEL: Terminal deoxynucleotidyl transferase-mediated dUTP-biotin nick and labeling
