# Supplementary material for: Drug-loaded microbubble delivery system to enhance PD-L1 blockade immunotherapy with remodeling immune microenvironment
Source: Biomater Res. 2023 Feb 9;27:9. doi: 10.1186/s40824-023-00350-5 (PMC9909878; doi:10.1186/s40824-023-00350-5)
Supplement: Supplementary file 1 — Additional file 1: Supporting Figure S1. Characterization of RD@MBs. (a) Light microscopy image of RD@MBs (scale bar = 10 μm). (b-c) The standard curves of DTX and R837 measured by LC-MS. Supporting Figure S2. Biosafety of RD@MBs. (a) Cell viabilities of 4T1 cells treated with different concentrations of RD@MBs (without US irradiation) (n = 3). (b) Heatmap of the blood biochemical and blood routine analysis (n = 5). (c) H&E images of major organs (heart, liver, spleen, lung and kidney) treated with different concentrations of RD@MBs for in vivo biosafety evaluation. Data are expressed as mean ± SD. Supporting Figure S3. UTMD drug delivery system. (a-b) In vitro US imaging and the corresponding echo intensities of RD@MBs at various concentrations (20, 40, 60, 80, 100 µg/mL) (n = 3). (c-d) In vivo US imaging and corresponding echo intensities of tumor regions at different time points (n = 3). Supporting Figure S4. UTMD drug delivery system. (a-b) FCM results of drug release in tumor sites and the corresponding quantitative analysis of MFI. Supporting Figure S5. Cell viabilities of 4T1 cells treated with different concentrations of R@MBs, D@MBs and RD@MBs (n = 3). Data are expressed as mean ± SD. Statistical significances were calculated via Student’s t test, *p < 0.05, **p < 0.01 and ***p < 0.001. Supporting Figure S6. (a-b) Primary and distant tumor TGI after different treatments in 4T1 orthotopic tumor bearing mice (n = 5). (c-d) Representative digital photos of tumor nodules in the 4T1 orthotopic tumor bearing mice lungs and corresponding quantification of the numbers of lung nodules (n = 5). (e) H&E and TUNEL staining images of primary tumor in 4T1 orthotopic tumor bearing mice. PCNA staining images of distant tumor in 4T1 orthotopic tumor bearing mice. (Scale bar = 200 μm). (f) Body weight of the 4T1 orthotopic tumor bearing mice after different treatments (n = 5). (g-h) Primary and distant tumor TGI after different treatments in CT26 subcutaneous tumor bearing m [file 40824_2023_350_MOESM1_ESM.docx]

***Supporting Information for***

**Drug-loaded Microbubble Delivery System to Enhance PD-L1 Blockade Immunotherapy with Remodeling Immune Microenvironment**

Jun Zheng^1 †^, Ju Huang^1 †^, Liang Zhang^1, 2*^, Mengna Wang^3^, Lihong Xu^4^, Xiaoyun Dou^4^, Xiaojing Leng^1^, Mingxiao Fang^1^, Yang Sun^1*^, Zhigang Wang^1*^

^1^State Key Laboratory of Ultrasound in Medicine and Engineering, Institute of Ultrasound Imaging, The Second Affiliated Hospital, Chongqing Medical University, Chongqing, 400010, PR China

^2^Ultrasound Department, The First Affiliated Hospital of Chongqing Medical University, Chongqing 400042, P. R. China.

^3^Department of Pathology, College of Basic Medicine, Chongqing Medical University, Chongqing, 400016, PR China.

^4^Institute of Life Sciences, Chongqing Medical University, Chongqing, 400016, PR China.

^*^Corresponding author: L. Zhang ([zhangliang338@cqmu.edu.cn](mailto:zhangliang338@cqmu.edu.cn)); Y. Sun ([sunyang@cqmu.edu.cn](mailto:sunyang@cqmu.edu.cn)); Z. Wang ([wangzhigang@cqmu.edu.cn](mailto:wangzhigang@cqmu.edu.cn))


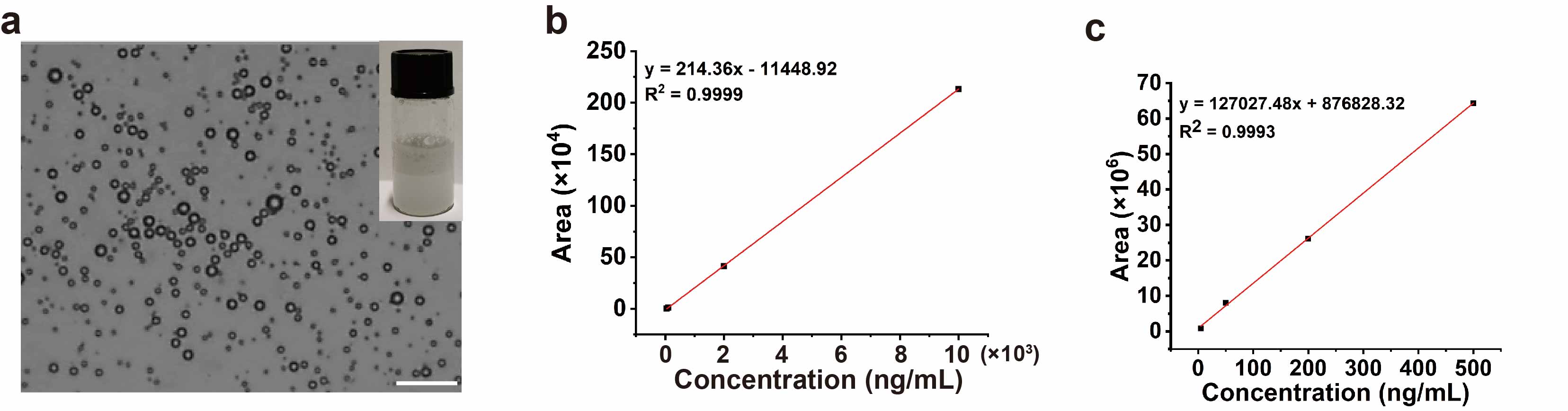


**Supporting Figure S1.** Characterization of RD@MBs. (a) Light microscopy image of RD@MBs (scale bar = 10 μm). (b-c) The standard curves of DTX and R837 measured by LC-MS.


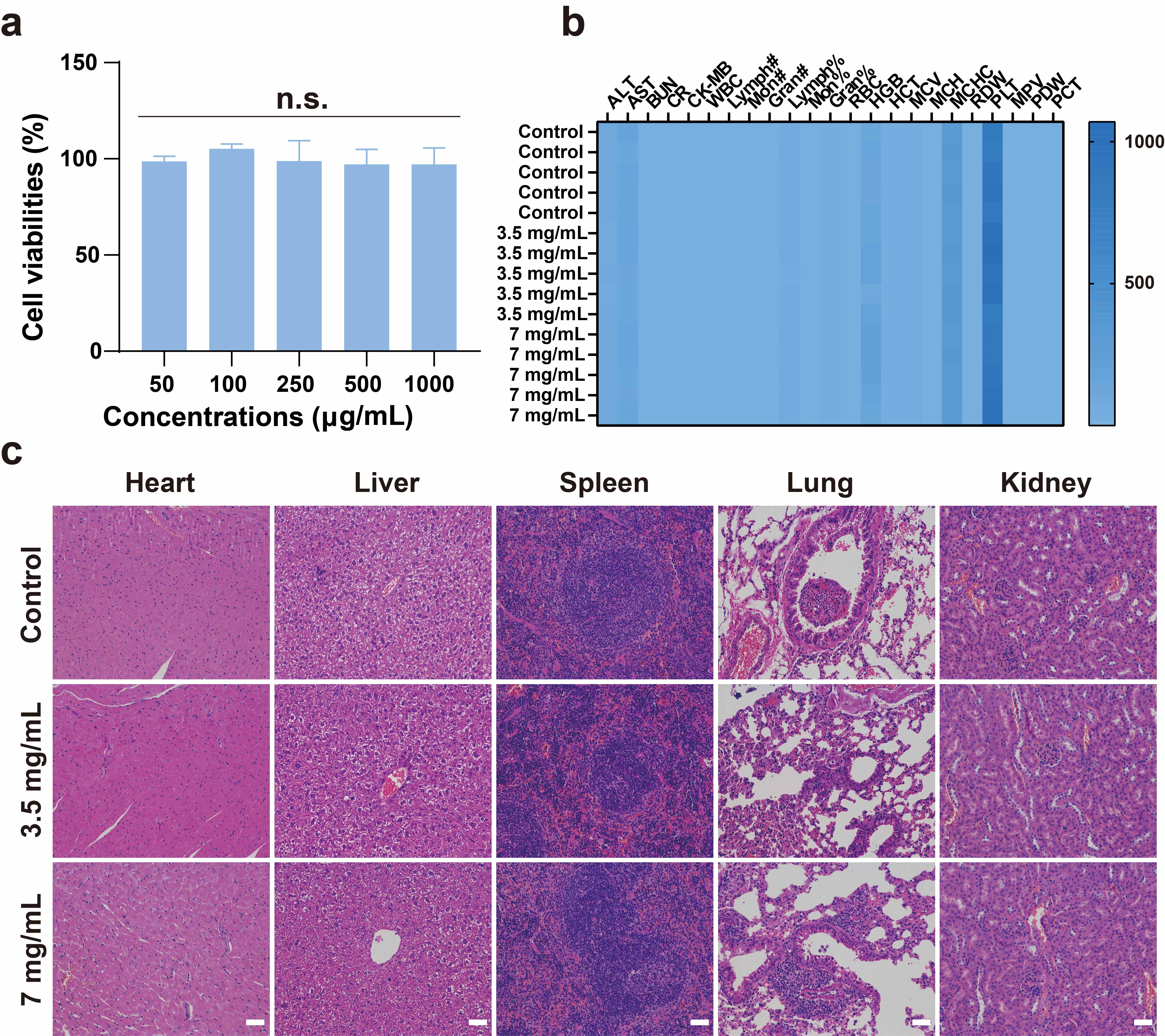


**Supporting Figure S2.** Biosafety of RD@MBs. (a) Cell viabilities of 4T1 cells treated with different concentrations of RD@MBs (without US irradiation) (n = 3). (b) Heatmap of the blood biochemical and blood routine analysis (n = 5). (c) H&E images of major organs (heart, liver, spleen, lung and kidney) treated with different concentrations of RD@MBs for *in vivo* biosafety evaluation. Data are expressed as mean ± SD.


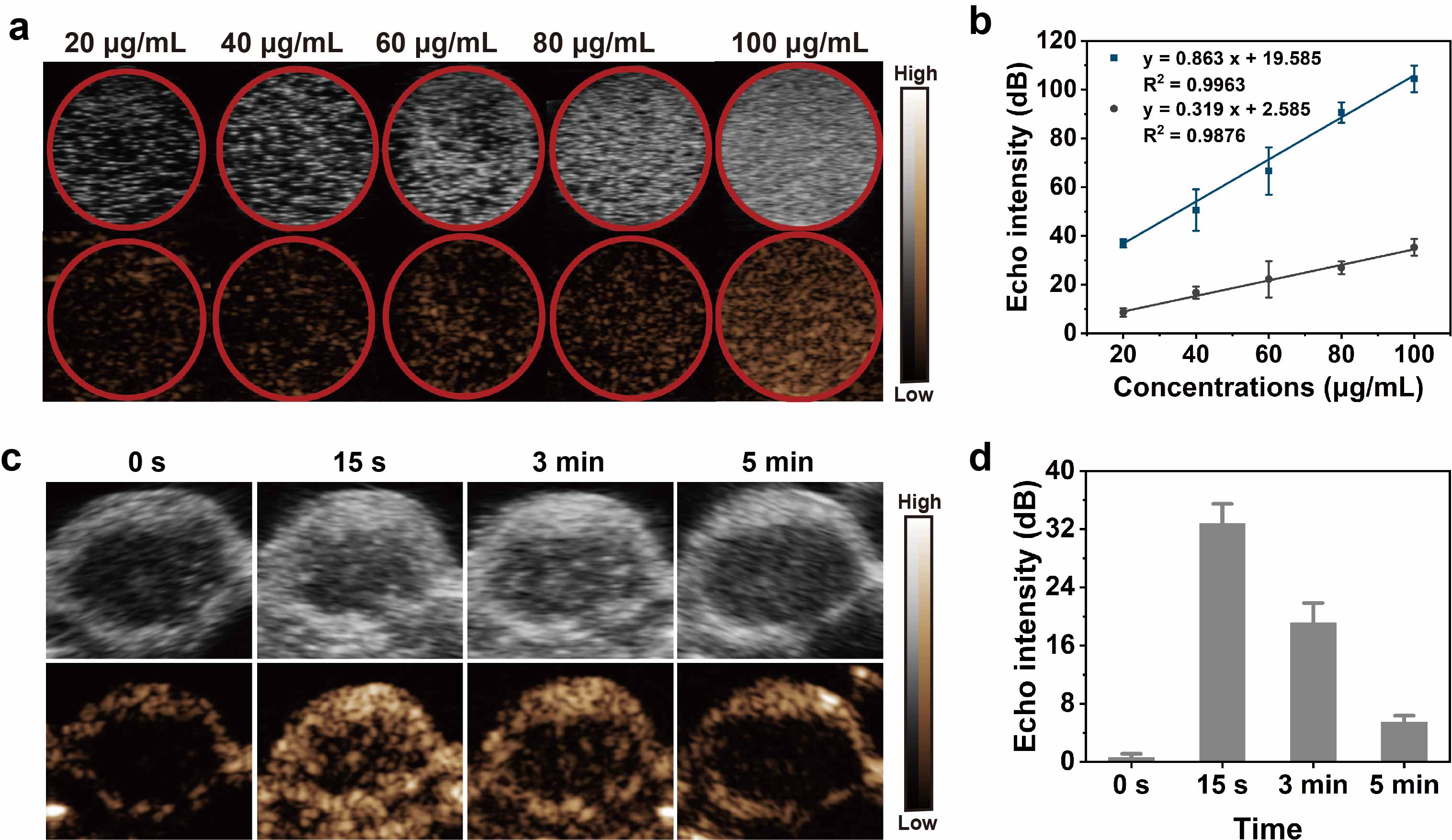


**Supporting Figure S3.** UTMD drug delivery system. (a-b) *In vitro* US imaging and the corresponding echo intensities of RD@MBs at various concentrations (20, 40, 60, 80, 100 µg/mL) (n = 3). (c-d) *In vivo* US imaging and corresponding echo intensities of tumor regions at different time points (n = 3).


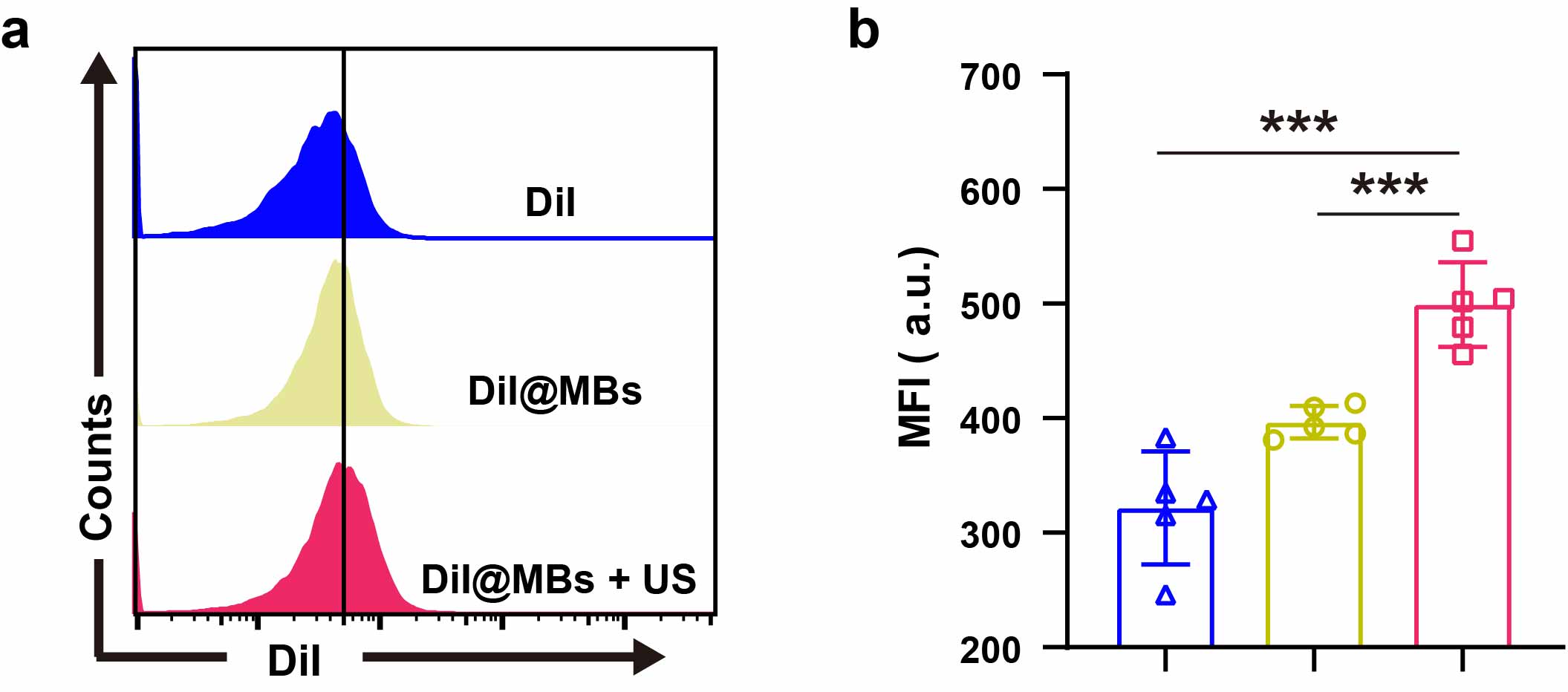


**Supporting Figure S4.** UTMD drug delivery system. (a-b) FCM results of drug release in tumor sites and the corresponding quantitative analysis of MFI.

**
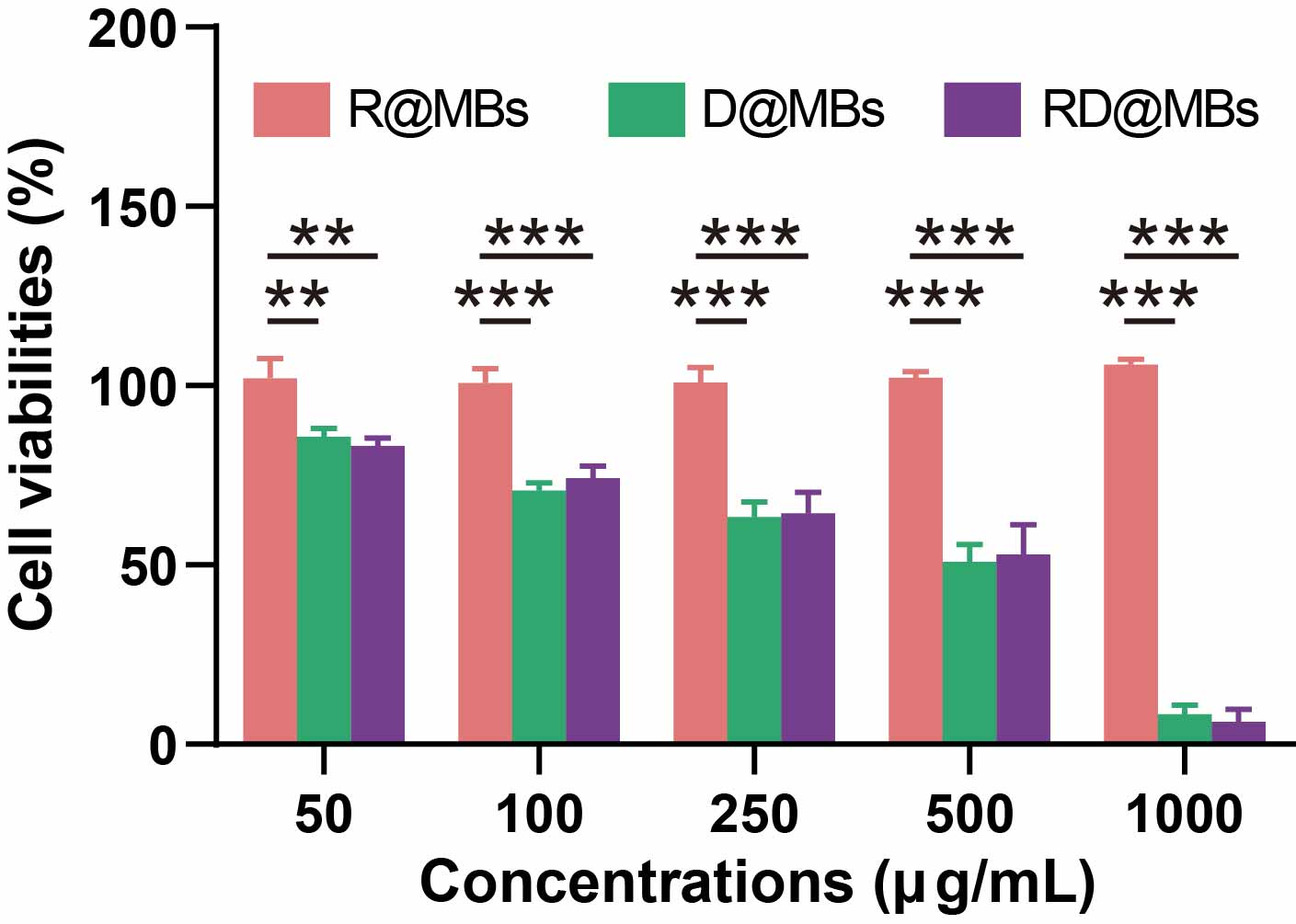
**

**Supporting Figure S5.** Cell viabilities of 4T1 cells treated with different concentrations of R@MBs, D@MBs and RD@MBs (n = 3). Data are expressed as mean ± SD. Statistical significances were calculated *via* Student’s t test, *p < 0.05, **p < 0.01 and ***p < 0.001.


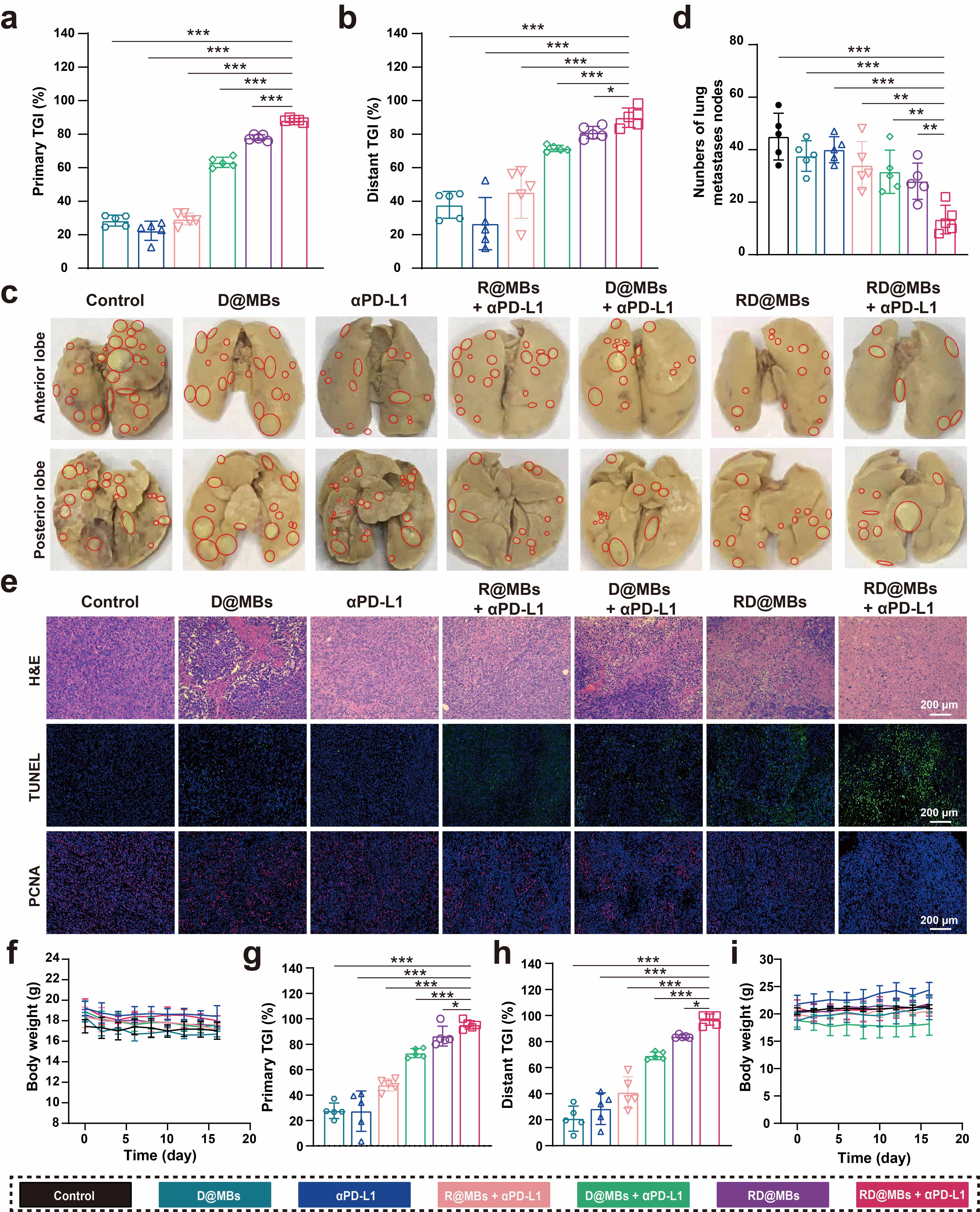


**Supporting Figure S6.** (a-b) Primary and distant tumor TGI after different treatments in 4T1 orthotopic tumor bearing mice (n = 5). (c-d) Representative digital photos of tumor nodules in the 4T1 orthotopic tumor bearing mice lungs and corresponding quantification of the numbers of lung nodules (n = 5). (e) H&E and TUNEL staining images of primary tumor in 4T1 orthotopic tumor bearing mice. PCNA staining images of distant tumor in 4T1 orthotopic tumor bearing mice. (Scale bar = 200 μm). (f) Body weight of the 4T1 orthotopic tumor bearing mice after different treatments (n = 5). (g-h) Primary and distant tumor TGI after different treatments in CT26 subcutaneous tumor bearing mice (n = 5). (i) Body weight of the CT26 subcutaneous tumor bearing mice after different treatments (n = 5). Data are expressed as mean ± SD. Statistical significances were calculated *via* Student’s t test, *p < 0.05, **p < 0.01 and ***p < 0.001.


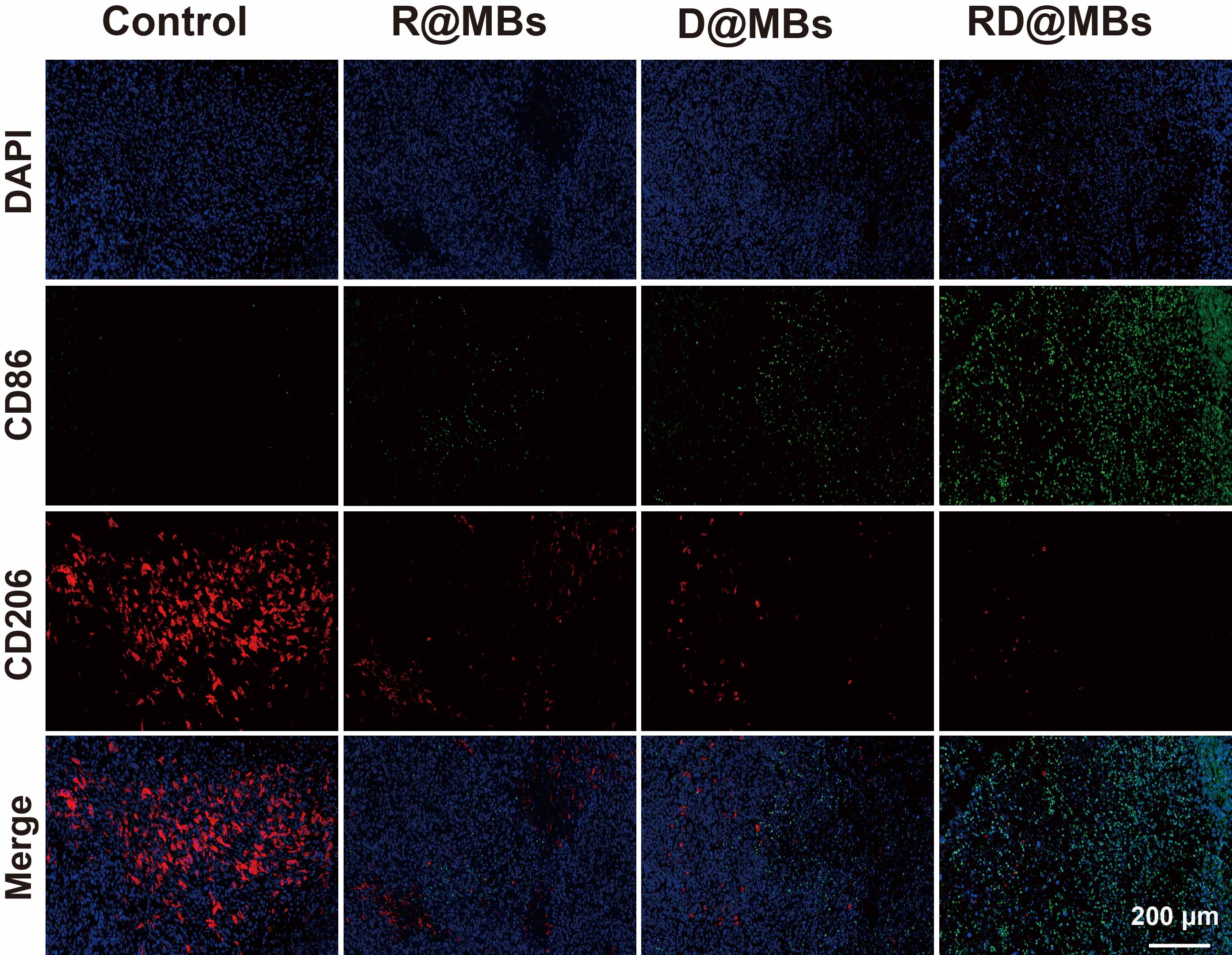


**Supporting Figure S7.** Immunofluorescence staining images for expression of CD86 and CD206 in 4T1 tumor tissues after different treatments. Blue, DAPI-labeled nucleus; green, anti-CD86 antibody-labelled M1-TAMs; red, anti-CD206 antibody-labeled M2-TAMs. (Scale bar = 200 μm).


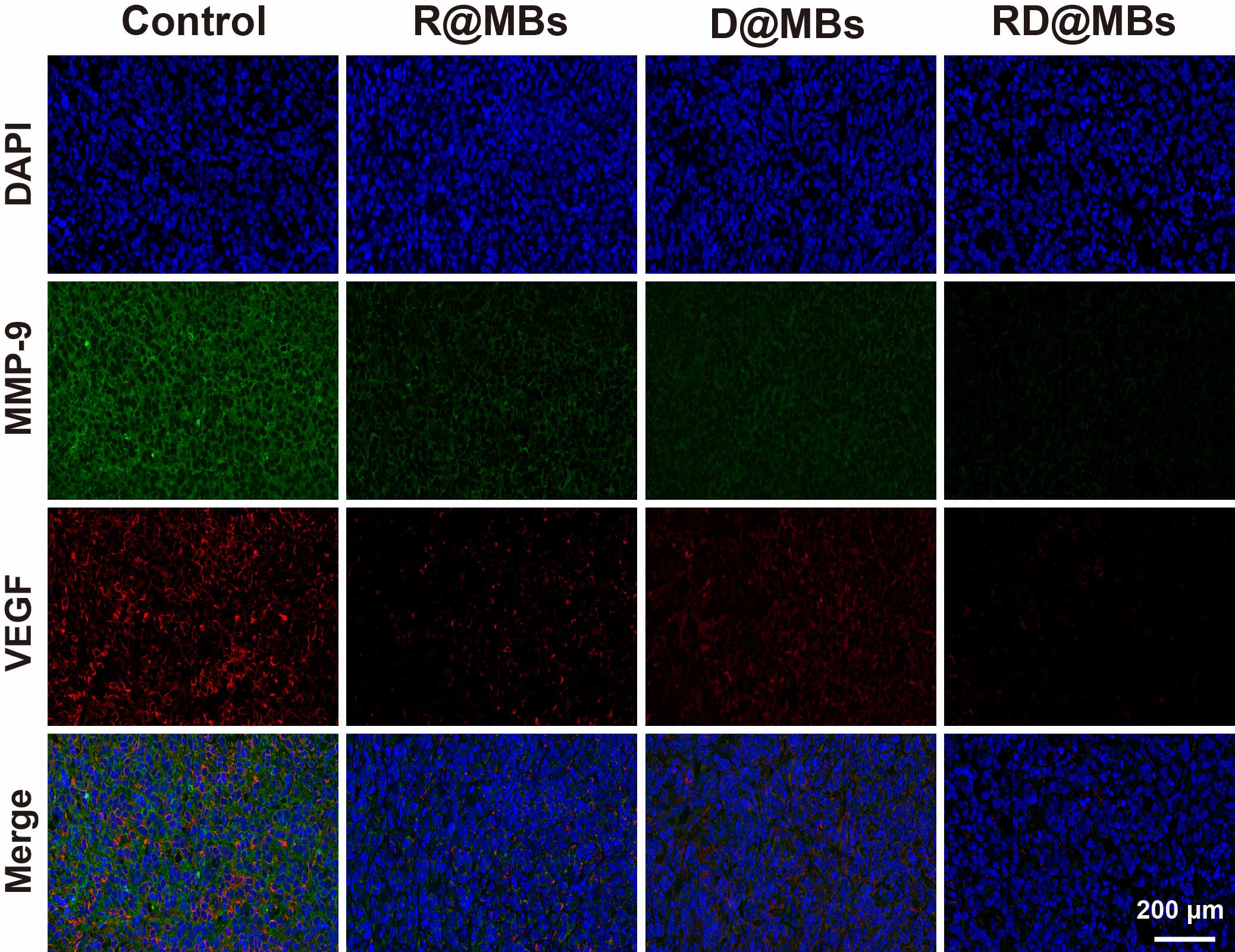


**Supporting Figure S8.** Immunofluorescence staining images for expression of MMP-9 and VEGF in 4T1 tumor tissues after different treatments. Blue, DAPI-labeled nucleus; green, anti-MMP-9 antibody-labelled MMP-9; red, anti-VEGF antibody-labeled VEGF (scale bar = 200 μm).


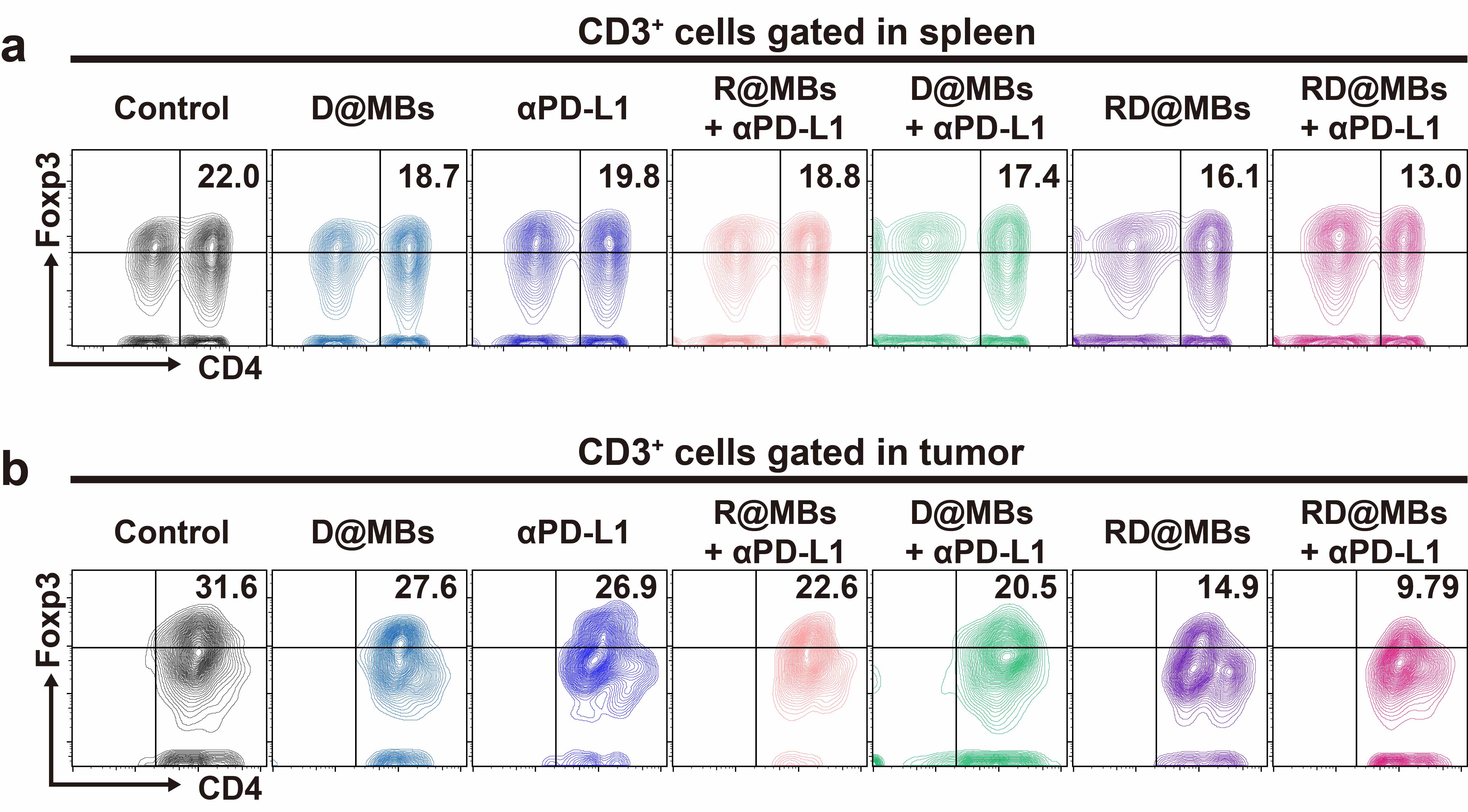


**Supporting Figure S9.** (a) FCM results of Tregs (CD3^+^CD4^+^Foxp3^+^) in spleen. (b) FCM results of Tregs (CD3^+^CD4^+^Foxp3^+^) in tumor.


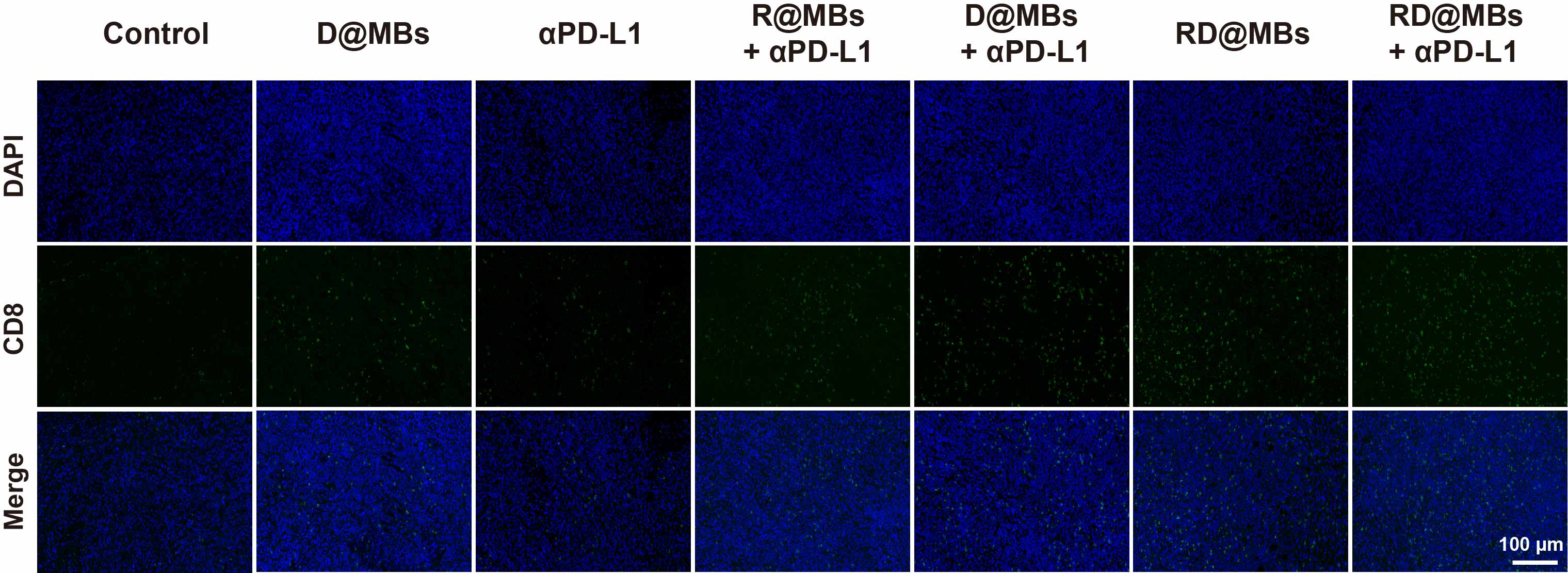


**Supporting Figure S10.** Immunofluorescence staining images for infiltration of CTLs in primary tumor of 4T1 orthotopic tumor bearing mice after different treatments. Blue, DAPI-labeled nucleus; green, anti-CD8 antibody-labelled CTLs (scale bar = 100 μm).
